# Bipolar membrane electrolyzers for co-upgrading of CO_2_ capture solutions and sulfide contaminants to syngas and sulfur

**DOI:** 10.1093/nsr/nwaf504

**Published:** 2025-11-19

**Authors:** Weisheng Yu, Fen Luo, Xian Liang, Xiaojiang Li, Wenfeng Li, Jingjing Tu, Luxin Xiong, Jihao Zhang, Liang Wu, Tongwen Xu

**Affiliations:** State Key Laboratory of Precision and Intelligent Chemistry, Department of Applied Chemistry, School of Chemistry and Materials Science, University of Science and Technology of China, Hefei 230026, China; State Key Laboratory of Precision and Intelligent Chemistry, Department of Applied Chemistry, School of Chemistry and Materials Science, University of Science and Technology of China, Hefei 230026, China; State Key Laboratory of Precision and Intelligent Chemistry, Department of Applied Chemistry, School of Chemistry and Materials Science, University of Science and Technology of China, Hefei 230026, China; State Key Laboratory of Precision and Intelligent Chemistry, Department of Applied Chemistry, School of Chemistry and Materials Science, University of Science and Technology of China, Hefei 230026, China; State Key Laboratory of Precision and Intelligent Chemistry, Department of Applied Chemistry, School of Chemistry and Materials Science, University of Science and Technology of China, Hefei 230026, China; State Key Laboratory of Precision and Intelligent Chemistry, Department of Applied Chemistry, School of Chemistry and Materials Science, University of Science and Technology of China, Hefei 230026, China; State Key Laboratory of Precision and Intelligent Chemistry, Department of Applied Chemistry, School of Chemistry and Materials Science, University of Science and Technology of China, Hefei 230026, China; State Key Laboratory of Precision and Intelligent Chemistry, Department of Applied Chemistry, School of Chemistry and Materials Science, University of Science and Technology of China, Hefei 230026, China; State Key Laboratory of Precision and Intelligent Chemistry, Department of Applied Chemistry, School of Chemistry and Materials Science, University of Science and Technology of China, Hefei 230026, China; State Key Laboratory of Precision and Intelligent Chemistry, Department of Applied Chemistry, School of Chemistry and Materials Science, University of Science and Technology of China, Hefei 230026, China

**Keywords:** bipolar membranes, paired electrolyzers, CO_2_ capture solutions electrolysis, sulfion oxidation, self-sustained system

## Abstract

Direct electrolysis of CO_2_ capture solutions (e.g. (bi)carbonate) streamlines upstream carbon supply, yet faces challenges including high cell voltage, low-value anode byproduct, and gaseous product impurity owing to incomplete CO_2_ utilization. Herein, we demonstrate a bipolar membrane (BPM) electrolyzer coupling CO_2_ capture solution reduction with sulfion oxidation reaction (SOR) for cogeneration of syngas and sulfur. Tailoring BPMs with rapid water dissociation kinetics and mass transfer facilitates paired reactions through pH gradients, with cathode acidification triggering *in situ* CO_2_ production for electroreduction while sustaining the alkaline environment necessary for anodic SOR. Leveraging gas–liquid extraction between the cathodic product stream and anolyte enables simultaneous syngas purification and sulfur precipitation, establishing a self-sustained system. With these material and process innovations, the paired electrolyzer achieves low energy consumptions (cell voltage <2.5 V), high carbon utilization (>97%), and long-term stable operation (>300 h) at 100 mA cm^−2^, continuously producing syngas (CO/H_2_ ratios = 2/1–1/1, with CO_2_ content <3%) and pure elemental sulfur.

## Teaser

This study pioneers a bipolar membrane-based electrolyzer that couples CO_2_ electroreduction with sulfur oxidation to simultaneously produce syngas and elemental sulfur from waste streams.

## INTRODUCTION

The electrochemical CO_2_ reduction reaction (CO_2_RR) powered by renewable energy offers a viable pathway for converting greenhouse gases into carbon-based chemicals and fuels [1,2]. Currently, the membrane electrode assembly (MEA)-integrated electrolyzers have significantly improved CO_2_ electrolysis efficiency and process operability [3,4]. However, mainstream CO_2_ gas-fed MEA electrolyzers suffer from limited long-term stability due to persistent challenges such as salt precipitation and (bi)carbonate crossover [5–8], falling short of practical application requirements. Furthermore, industrial-scale implementation remains hindered by cumbersome upstream feedstock supply and downstream separation processes, which constitute >50% of the total operating costs [9–11].

Direct electrolysis of CO_2_ capture solutions is gaining attention as a promising alternative for conventional gaseous feedstocks (Fig. S1) [12,13]. This method employs a bipolar membrane (BPM) under reverse bias to drive water dissociation (H_2_O → H^+^ + OH^−^), with the resulting protons (H^+^) migrating to the cathode to react with (bi)carbonates, enabling *in situ* CO_2_ (*i*-CO_2_) generation for subsequent CO_2_RR (Fig. S2) [14,15]. Crucially, this integrated approach eliminates energy-intensive processes of CO_2_ desorption and gas concentration, thereby effectively bridging the gap between carbon capture and utilization [16,17]. Moreover, liquid-based feedstock addresses the salt precipitation issue, while the BPM reverse bias configuration avoids (bi)carbonate crossover through combined electroosmotic resistance and Donnan exclusion [18,19]. Despite demonstrating superior process operability and stability, (bi)carbonate electrolyzers still face significant challenges. First, the BPM requires additional energy input to drive water dissociation, combining with intrinsically sluggish kinetics of the anode oxygen evolution reaction (OER), and resulting in high cell voltages of ~3.5–5.0 V [20]. Second, the low-value anode products (O_2_) undermine the economic viability of the overall process [21]. Furthermore, incomplete conversion of *i*-CO_2_ (40%–80%) at the cathode introduces gaseous impurities, necessitating energy-intensive purification processes [22].

Electrochemical coupling has been proposed to substitute OER with various electrooxidation reactions. This approach simultaneously enhances energy efficiency and economic viability, while in some instances, circumventing detrimental electrode reactions [23–25]. For instance, coupling the hydrogen oxidation reaction (HOR) with CO_2_RR can reduce the electrolyzer energy penalty by 42% [26], while incorporating electrooxidation of polyols and biomass not only substantially lowers cell voltage but also enables value-added conversion [27–30]. Notably, most electrooxidation reactions favor alkaline conditions for enhanced kinetics—a feature well-aligned with the water dissociation configuration of BPM electrolyzers. As exemplified by the sulfion oxidation reaction (SOR), alkaline environments are a prerequisite for both stabilizing S^2−^ and facilitating the dissolution of oxidation products as polysulfides, thereby preventing electrode passivation by insoluble deposits [31,32]. Limited studies have attempted to electrochemically couple CO_2_RR with SOR, achieving notable reductions in energy consumption [33,34]. However, the use of monopolar membranes in these systems hindered reaction coordination between the anode and cathode, preventing sustained long-term operation. These insights suggest that BPMs can potentially address two fundamental challenges in paired electrolyzers: (1) simultaneously accommodating divergent pH requirements for paired electrode reactions, and (2) preventing species crossover via exact ionic segregation.

**Figure 1.**
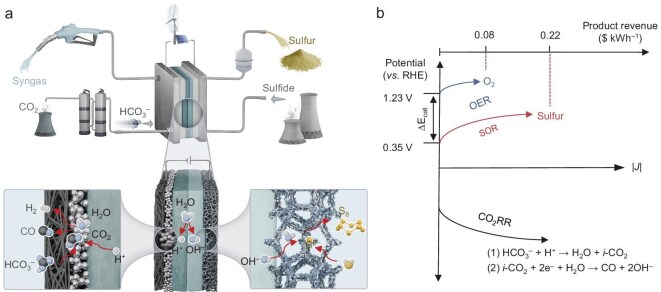
Design concept of the CO_2_RR|BPM|SOR paired electrolyzer. (a) Schematic illustration of the CO_2_RR|BPM|SOR paired electrolyzer. The electrolyzer employs a BPM to separate the cathode and anode. Under reverse bias, water dissociation at the BPM interface generates H^+^ that migrate to the cathode, acidifying bicarbonate to produce CO_2_ for subsequent reduction. Simultaneously, water dissociation creates an alkaline cathodic environment favorable for SOR. (b) Comparison of electrode potentials and product value-added between SOR and OER. SOR exhibits lower potential and is more economically favorable than OER.

Here, we design an electrochemically paired system using BPMs that integrates cathodic bicarbonate-based CO_2_RR with anodic SOR (CO_2_RR|BPM|SOR, Fig. 1a). This innovative system represents a multi-win strategy. The liquid-fed cathode simplifies feedstock supply while mitigating salt precipitation and bicarbonate crossover. Meanwhile, alkaline-mediated SOR at the anode simultaneously reduces cell voltage and enables value-added conversion of harmful sulfide pollutants into chemical elemental sulfur. Comparative experiments combined with numerical simulations elucidate the critical role of BPMs in regulating pH and addressing CO_2_-starvation within the paired electrolyzers. However, commercial BPMs have limited practical operation due to inefficient water dissociation and suboptimal mass transport properties. We therefore adopt tailored BPMs to expand the operational window to industrial-grade current densities. Moreover, in leveraging CO_2_-induced acidification of the anolyte, we established a self-sustaining cycle. This system integrates gas–liquid extraction between the cathode outlet stream and the anolyte, simultaneously purifying the cathode syngas and precipitating sulfur as the anode product. Energy consumption and carbon emission analyses reveal that our paired electrolyzer offers superior energy-saving and emission-reduction characteristics compared to conventional (bi)carbonate- and CO_2_ gas-fed electrolyzers.

## RESULTS AND DISCUSSION

### Limitations in monopolar membrane-integrated systems

SOR is employed to substitute OER in paired electrolyzers due to its substantially lower thermodynamic potential (0.14 and 1.23 V vs reversible hydrogen electrode (RHE) at pH = 14), higher value of electrochemical product (0.22 and 0.08$ kWh^−1^ for sulfur and O_2_) (Fig. 1b and Fig. S3), and environmental merits in treating sulfide contaminants [35,36]. Previous studies on SOR have primarily focused on designing efficient catalysts, offering valuable guidance for the preparation of anode materials in the present work. Following previously reported procedures, we selected cobalt-based sulfides as the model catalyst and prepared nickel (Ni) foam-supported Co_3_S_4_ (Co_3_S_4_@Ni foam) via a two-step hydrothermal method (see Methods for details) [37,38]. Figures S4–S6 present the scanning electron microscope (SEM) and corresponding energy-dispersive X-ray spectrometry (EDX) mapping images of bare Ni foam, Ni foam-supported Co(OH)_2_ precursor (Co(OH)_2_@Ni foam), and Co_3_S_4_@Ni foam, respectively. Co(OH)_2_ exhibits a needle-like morphology, whereas Co_3_S_4_ shows a nanocluster structure. Their phase structures were further confirmed by the X-ray diffraction (XRD) patterns (Fig. S7).

We evaluated the electrocatalytic performance of the as-prepared Co_3_S_4_@Ni foam for SOR and OER in a standard three-electrode system, with the blank controls (Ni foam) and commercial Pt–Ru/C-coated Ni foam (Fig. S8, see Supplementary Methods for detailed preparation) tested for comparison. Figure S9a and b present their linear sweep voltammetry (LSV) curves for OER and SOR, respectively. In agreement with previous reports [37,38], cobalt-based sulfide electrodes exhibit relatively superior SOR catalytic activity compared to bare Ni foam and Pt–Ru/C@Ni foam. Notably, the incorporation of SOR significantly reduces the electrode potential, evidenced by an ~1.0 V decrease in onset potential compared to OER (from 1.32 to 0.31 V vs RHE at 10 mA cm^−2^, Fig. S9c). During electrolysis, the electrolyte solution transitioned from colorless to yellow with progressive darkening (Fig. S10a). UV spectrophotometric methods confirmed that this color evolution was attributable to the advancement of SOR, as verified by the characteristic absorption of polysulfide intermediates (S_2_^2−^–S_4_^2−^) at 300 and 370 nm (Fig. S10b). For cathodic (bi)carbonate electrolysis, Ag nanoparticle-coated hydrophilic carbon paper (Ag@carbon paper) served as an efficient gas diffusion electrode (GDE) (Fig. S11, fabricated as described in the Methods).

**Figure 2.**
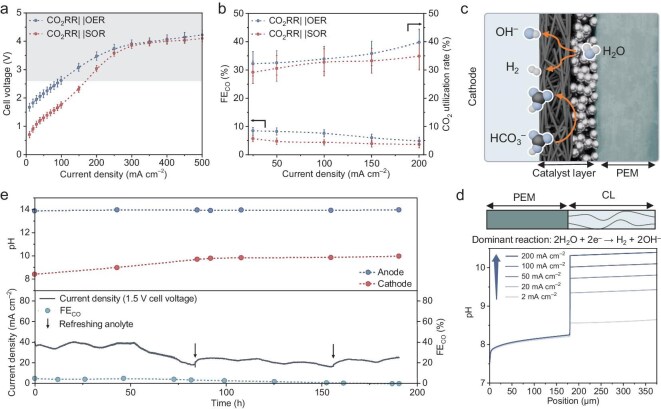
Paired electrolyzer performed with PEM. (a) Polarization curves of the CO_2_RR||SOR and CO_2_RR||OER electrolyzers equipped a Nafion 117 PEM. The CO_2_RR||SOR electrolyzer requires a lower voltage than CO_2_RR|| OER below 200 mA cm^−2^, but the voltage convergence at higher current densities indicate a transition from SOR to OER as the dominant anodic reaction. (b) Current density-dependent FE_CO_ and CO_2_ utilization rate of the electrolyzers. (c) Schematic illustration of the main reactions at the cathode, where HER prevails as the main pathway with H_2_ production under CO_2_-starved conditions. (d) Modeled pH distribution within the cathode for different applied current densities. (e) Evolution of current density, FE_CO_, and electrode pH values of the CO_2_RR||SOR electrolyzer during stability testing at a cell voltage of 1.5 V.

We assembled a bicarbonate reduction and SOR paired electrolyzer using a Nafion 117 (with a thickness of 180 µm) proton exchange membrane (CO_2_RR|PEM|SOR), with the CO_2_RR|PEM|OER system serving as the control group. Figure 2a presents the polarization curves of both electrolyzers at room temperature, revealing that the CO_2_RR|PEM|SOR exhibited significantly lower cell voltage compared to the OER-integrated system (e.g. 1.75 vs 2.61 V at 100 mA cm^−2^). Notably, we observed that the CO_2_RR|PEM|SOR electrolyzer operated optimally within a specific current density window (0–200 mA cm^−2^). Exceeding this window resulted in cell voltage elevation approaching that of the CO_2_RR|PEM|OER system, which we attribute to the predominance of competitive OER at higher potentials. Moreover, both OER- and SOR-involved electrolyzers exhibited relatively low CO generation Faradaic efficiency (FE_CO_) and CO_2_ utilization rate, remaining below 10% and 40% across the current density range of 0–200 mA cm^−2^ (Fig. 2b).

To elucidate the underlying mechanism of the low electrolysis efficiency in PEM-integrated systems, we conducted numerical simulations (Fig. S12a, see Supplementary Methods for details). Building upon the work of Weng *et al.* and Lees *et al.* [39,40], we established a one-dimensional continuum model to study reaction and mass transport behaviors in the cathode compartment (Fig. 2c). In the PEM-integrated system, the hydrogen evolution reaction (HER) dominates in the cathode chamber. The concomitant generation of hydroxide ions (OH^−^) elevates the local pH, which suppresses *i*-CO_2_ formation due to the pH-dependent speciation of (bi)carbonate species (Fig. S13). As shown in Fig. 2d, the cathode pH increases progressively with rising HER current density. This inherent alkaline local environment creates a CO_2_-starved condition, accounting for the low CO_2_RR Faradaic efficiency [40]. To validate the modeling results, we performed an electrolysis stability test at a constant applied voltage of 1.5 V for ~200 h. As illustrated in Fig. 2e, the current density initially remained stable at ~40 mA cm^−2^ but exhibited progressive decay during prolonged operation. Although electrolyte replenishment was implemented to address continuous species consumption, only partial current density was resumed. The pH variation at both electrodes during electrolysis was monitored. Due to proton consumption induced by HER, the cathode pH rapidly increased from 8.4 to 9.7 within 85 h, creating local alkaline environments incapable of protonating bicarbonate. These results aligned well with numerical simulations. Moreover, the detection of sulfide at the cathode suggests incomplete blocking of active species migration by PEM alone (Fig. S14c). We therefore propose that the irreversible cell performance decay is partially associated with crossover-induced contamination.

### Bipolar membranes enable efficient paired electrolysis

**Figure 3.**
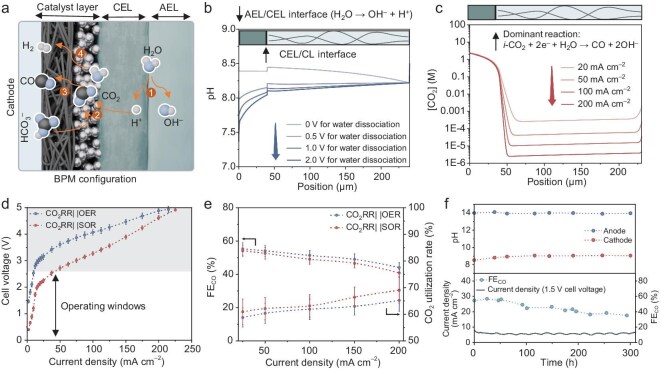
Paired electrolyzer performed with commercial BPM. (a) Schematic illustration of the main reactions at the cathode. H^+^ from the BPM interface migrate to the cathode (step 1), reacting with bicarbonate to generate CO_2_ (step 2), which is subsequently reduced to CO (step 3) alongside competitive HER (step 4). (b) Modeled pH distribution within the cathode for different water dissociation voltages. (c) Modeled CO_2_ concentration within the cathode for different CO_2_RR current densities. (d) Polarization curves of the CO_2_RR||SOR and CO_2_RR||OER electrolyzers equipped with a commercial FBM-PK BPM. Commercial membranes exhibit low water dissociation efficiency and slow mass transport, resulting in high cell voltages that limit the effective current density window to below 50 mA cm^−2^. (e) Current density-dependent FE_CO_ and CO_2_ utilization rate of the electrolyzers. (f) Evolution of current density, FE_CO_, and electrode pH values of the CO_2_RR||SOR electrolyzer during stability testing at a cell voltage of 1.5 V.

The BPM-integrated configuration could theoretically address the aforementioned intrinsic issues in PEM systems. Research by Berlinguette’s group and Sargent’s group has systematically demonstrated the role of BPMs in (bi)carbonate electrolyzers [41–43]. As illustrated in Fig. 3a, under reverse bias, water dissociation at the BPM interface generates H^+^ that migrate toward the cathode (step 1), acidifying (bi)carbonate and enabling *i-*CO_2_ generation (step 2), which subsequently undergoes electroreduction on the electrode (step 3). To validate this, we computationally modeled the species distribution in this architecture. We first simulated the pH variation induced by water dissociation at different voltages. As illustrated in Fig. 3b, the cathodic pH in the BPM system is significantly lower than that in a PEM system due to H^+^ generation from water dissociation. Increasing the water dissociation voltage enhances the H^+^ flux, leading to a more pronounced pH drop. The lowest pH occurs near the bipolar junction, gradually increasing with distance from the interface, indicating H^+^ consumption via bicarbonate acidification [37]. The HCO_3_^−^ concentration profile in Fig. S15a demonstrates that the decreasing trend becomes more pronounced with increasing proton generation rate from water dissociation. This acidification-induced HCO_3_^−^ depletion results in significantly lower concentrations near the BPM compared to PEM (Fig. S15b). Subsequently, we simulated the CO_2_ concentration distribution at the cathode side under fixed water dissociation voltage. Figure 3c presents the CO_2_ concentration profiles at different CO_2_RR current densities. Notably, higher CO_2_ concentrations are observed within the cation exchange layer (CEL) due to bicarbonate acidification by H^+^, while a significant drop in concentration occurs at the catalyst layer (CL)/CEL interface owing to CO_2_ consumption via electrochemical reduction. This depletion effect becomes more pronounced with increasing CO_2_ reduction current density. These observations align well with previous modeling studies [40], theoretically validating the essential role of BPM water dissociation in enabling efficient (bi)carbonate electrolysis.

A commercial BPM (Fumasep FBM-PK, with a thickness of ~130 µm) was employed to assemble the paired electrolyzer (CO_2_RR|BPM|SOR) for performance evaluation, with a CO_2_RR|BPM|OER configuration serving as the control group. The polarization curves in Fig. 3d demonstrate that the CO_2_RR|BPM|SOR configuration effectively reduces cell voltage compared to the OER-involved counterpart, thereby decreasing energy consumption—similar to the PEM system. Notably, the incorporation of BPM significantly enhances the CO_2_RR efficiency. As shown in Fig. 3e, the CO_2_RR|BPM|SOR electrolyzer achieves a FE_CO_ of 40%–55% and a carbon utilization rate of 60%–70% at current densities ranging from 0 to 200 mA cm^−2^, with the CO_2_RR|BPM|OER system performing similarly. This is in agreement with COMSOL simulations, which confirm the unique advantages of BPMs in (bi)carbonate electrolyzers. Subsequently, we conducted a stability test under a constant cell voltage of 1.5 V for over 300 h, while monitoring the electrolyte pH evolution during electrolysis (Fig. 3f). The FE_CO_ declined progressively from 55% to 36% over 300 h of operation, attributable to carbon source depletion in the catholyte. Unlike the PEM-integrated system, the BPM electrolyzer exhibited negligible pH elevation from 8.53 to 9.07 in the catholyte. Combined experimental and modeling analyses revealed that this phenomenon stems from improved CO_2_RR performance, which effectively suppresses competing HER and parasitic OH^−^ generation. Furthermore, the BPM configuration more effectively prevented crossover of active species between electrodes (Fig. S14d). Combining pH regulation and active-species blocking, the BPM electrolyzer demonstrates enhanced stability, as evidenced by the gradual current density decay rate. While commercial BPMs improved both electrolysis efficiency and stability, they simultaneously increased the overall cell voltage. Compared to PEM systems, the BPM configuration exhibited substantially lower current densities at equivalent cell voltages (<10 mA cm^−2^ at 1.5 V), with the operational current density window narrowing to below 50 mA cm^−2^ (Fig. 3d). The anode reactions at current densities below and above the operational window are presented in Videos S1 and S2, respectively. When exceeding the operational window, pronounced OER occurs, generating oxygen and consequently reducing the SOR efficiency. To further improve the energy efficiency and expand the operational window, engineering high-performance BPMs is critically required.

### Engineering bipolar membrane and system for sustainable paired electrolysis

**Figure 4.**
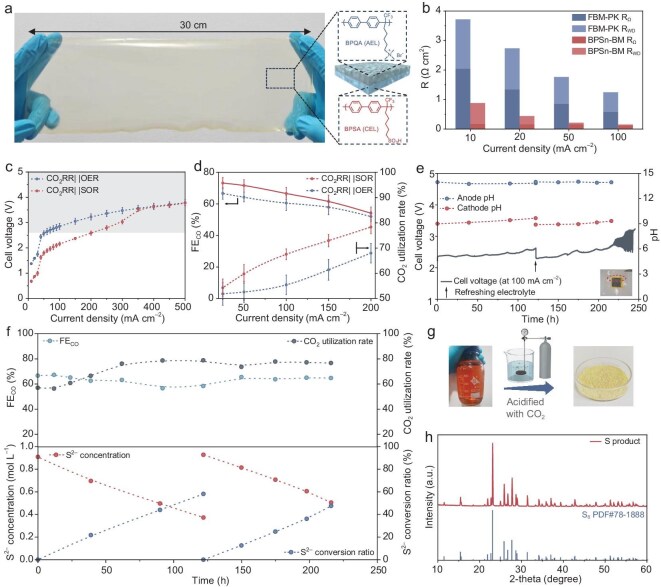
Paired electrolyzer performed with tailored BPM. (a) Structural authentication of BPSn-BM. Molecular formula and scale photograph (30 × 15 cm). (b) Ohmic and water dissociation resistances of the commercial FBM-PK and home-made BPSn-BM membranes for different current densities. (c) Polarization curves of the CO_2_RR||SOR and CO_2_RR||OER electrolyzers equipped a BPSn-BM BPM. The electrolyzer with advanced BPMs demonstrates significantly reduced cell voltage compared to commercial counterparts, thereby extending the current density window to 200 mA cm^−2^. (d) Current density-dependent FE_CO_ and CO_2_ utilization rate of the electrolyzers. Evolution of (e) cell voltage, electrode pH values and (f) FE_CO_, CO_2_ utilization rate, and anodic S^2−^ concentration of the CO_2_RR||SOR electrolyzer during stability testing at a current density of 100 mA cm^−2^. (g) CO_2_-mediated anolyte acidification process. (h) XRD pattern of the collected sulfur product with S_8_ reference standard (PDF#78–1888).

Sluggish water dissociation and suboptimal mass transfer are the primary factors restricting the application of commercial BPM electrolyzers [44–47]. Our previous work addressed these challenges by tailoring membrane molecular structures, screening high-efficiency water dissociation catalysts, and developing advanced fabrication processes, ultimately achieving high-performance and durable BPMs [48,49]. We propose that such engineered BPMs are applicable in the present study, which not only enhance mass transport within the MEA to promote dynamic reaction equilibrium, but their durability is also critical for the long-term operation of the paired electrolyzers. Building upon our foundational work (Note S1), we fabricated a BPM with a poly(biphenyl alkylene) backbone as illustrated in Fig. 4a, Figs S17 and S18 (BPSn-BM, with a thickness of ~50 µm). The fabricated anion- and cation-exchange layers (AEL and CEL) demonstrated robust mechanical properties with a fracture strength exceeding 40 MPa (Fig. S19a), providing essential physical strength to the BPMs. The exceptional ion and water transport properties fulfill rapid mass transfer requirements (Fig. S19b). Moreover, the chemically identical AEL and CEL ensures a robust bipolar interface (Fig. S20), exhibiting superior interfacial integrity and uniformity compared to commercial BPMs (Fig. S21). Given the potential for CO_2_ generation at the interface due to bicarbonate ion migration into the BPM (the CEL inherently cannot completely block anions), robust interfacial strength is crucial for ensuring long-term operational stability. Systematic electrochemical impedance spectroscopy (EIS) analysis quantified key water dissociation parameters (Note S2 and Fig. S23), revealing significantly reduced Ohmic resistance (R_Ω_) and interfacial water dissociation resistance (R_WD_) compared to commercial counterparts (Fig. 4b). These improvements reflect synergistic enhancements in both membrane layer mass transfer and interfacial water dissociation kinetics. As a result, the BPSn-BM demonstrates superior polarization behavior with a 0.31 V reduction in transmembrane voltage compared to commercial FBM-PK (0.73 vs 1.04 V at 100 mA cm^−2^, Fig. S24a) and other reported BPMs (Table S2). Remarkably, despite its reduced thickness, our BPM exhibits a lower first limiting current density (*I_lim1_*, 9.8 vs 16.7 mA cm^−2^), which is attributed to its exceptional co-ion rejection capability (Fig. S24b). This property is particularly advantageous for minimizing crossover of undesirable ionic species in paired electrolyzers.

Figure 4c demonstrates the polarization curves of the fabricated BPSn-BM in CO_2_RR||OER and CO_2_RR||SOR electrolyzers, which exhibits a significantly lower cell voltage than commercial BPMs, extending the effective operation window to over 200 mA cm^−2^. At industrially relevant current densities of 100 and 200 mA cm^−2^, the CRR||SOR electrolyzer demonstrates low cell voltages of 2.16 and 2.59 V. This performance surpasses the CO_2_RR||OER system (2.85 and 3.23 V) and most previously reported (bi)carbonate electrolyzers (Table S3), with only anode HOR-coupled (bi)carbonate electrolysis achieving comparable cell voltage (~2.3 V at 220 mA cm^−2^) [50]. The optimized BPM not only improves energy efficiency but also achieves superior FE and CO production (54%–73%) and CO_2_ utilization rate (56%–78%) across the operational current density range of 25–200 mA cm^−2^ (Fig. 4d). Combined model analysis and electrochemical performance studies reveal that enhancing the water dissociation efficiency and mass transfer of BPMs promotes localized acidification at the cathode, thereby creating a CO_2_-enriched environment and improving the selectivity of the CO_2_RR.

We evaluated the operational stability of the CO_2_RR||SOR electrolyzer at a current density of 100 mA cm^−2^. During the initial 200 h stability testing, the cell voltage exhibited only marginal increase (from 2.3 to 2.6 V) attributable to reactant depletion, which was fully recoverable through electrolyte replenishment (Fig. 4e). *In-operando* pH monitoring confirmed the critical role of the BPM configuration in maintaining dynamic pH equilibrium of the electrolyzer, with the cathode pH only increasing gradually from 9.0 to 9.7 after 120 h of continuous operation. Throughout the stability test, the cathode maintained consistent FE_CO_ (60% ± 5%) and high CO_2_ utilization rate (70% ± 10%) (Fig. 4f). Meanwhile, the S^2−^ concentration in the anolyte decreased linearly, and this trend repeated consistently after anolyte replacement (Fig. S25). Overall, our system demonstrated superior stability compared to previously reported (bi)carbonate electrolyzers. However, beyond 220 h of continuous operation, the cell voltage exhibited severe fluctuations followed by a rapid rise. Post-mortem analysis revealed catastrophic fracture of the cathode GDE (photos in Fig. 4e and Fig. S26), which we attribute to combined physical degradation of the carbon paper substrate from both fluid erosion and mechanical stress. It should be noted that although the commercial BPM electrolyzer appeared to operate continuously for a longer duration (Fig. 3f), it was run at an extremely low current density, where the limited electrode reaction kinetics did not generate enough substantial gas flow to cause damage to the GDE. A recent study demonstrated that gaseous CO_2_ can be employed as an acid source to acidify and precipitate SOR electrolytes [51]. This approach not only avoids additional sulfuric acid usage but also provides an efficient carbon capture and utilization strategy. Following the procedure illustrated in Fig. 4g and Fig. S27, we acidified the anolyte with CO_2_ (Video S3 demonstrates the introduction of CO_2_ into the anolyte via a gas diffuser), yielding a yellow powdered product. XRD patterns confirmed the crystalline powder to be phase-pure sulfur (S_8_, PDF#78–1888) (Fig. 4h).

**Figure 5.**
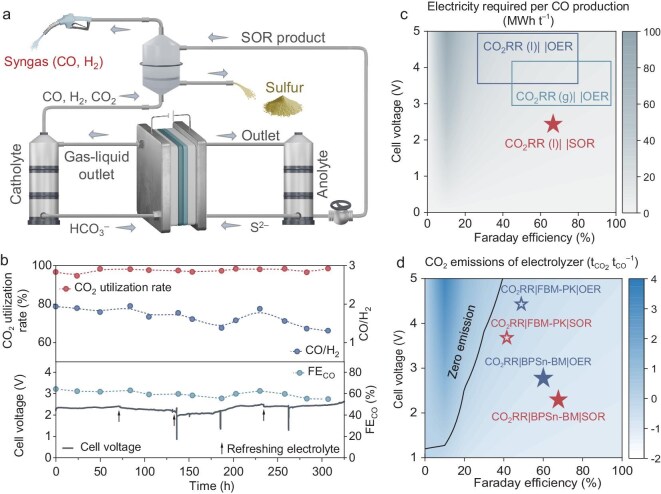
Self-sustained system design for the CO_2_RR||SOR paired electrolyzer. (a) Schematic illustration of the self-sustained CO_2_RR||SOR paired electrolyzer. Gas–liquid extraction between the cathode out stream and anolyte enables simultaneous cathodic syngas purification and anolyte acidic precipitation. (b) Evolution of cell voltage, FE_CO_, CO_2_ utilization rate, and CO/H_2_ ratio of the self-sustained electrolyzer during stability testing at a current density of 100 mA cm^−2^. (c) Energy consumption comparison of our paired electrolyzer versus conventional (bi)carbonate- and gaseous CO_2_-fed systems. (d) Carbon emission comparison of the optimized bicarbonate electrolyzer (CO_2_RR|BPSn-BM|SOR) versus commercial BPM-integrated (CO_2_RR|FBM-PK|SOR and CO_2_RR|FBM-PK|OER) and SOR-free configurations (CO_2_RR|BPSn-BM|OER).

Considering the acidifying effect of gas CO_2_ on the SOR anolyte, we propose redirecting the cathode outlet gas stream into the anolyte to establish a self-sustained system (Fig. 5a). This approach serves dual purposes: (1) sustainably extracting elemental sulfur from the anolyte, while (2) purifying CO_2_ in the cathode product gas stream, thereby enhancing product purity and CO_2_ utilization. To further improve the durability of the electrolyzer, optimization of the existing electrode materials is essential. Previous work by Zhang *et al.* demonstrated that porous Ag foam is a promising (bi)carbonate electrode, with its superior mechanical properties and ductility ensuring long-term stability [52]. Based on these design principles, we assembled the electrolyzer system, wherein Ag foam replaces Ag nanoparticles-based GDE (Fig. S28), while the recirculated cathode exhaust achieves self-purification through anolyte integration (Fig. S29 and Video S4). Compared to Ag@carbon paper, porous Ag foam shows negligible effects on both electrolyzer polarization and CO_2_RR performance (Fig. S30). Notably, however, the optimized system demonstrates stable operation for over 300 h (Fig. 5b), exhibiting minor CO_2_RR Faradaic efficiency decay (from 64% to 55%) and cell voltage increase (from 2.3 to 2.6 V, with transient spikes arising from the formation and subsequent dispersal of blockages caused by gaseous reactants and products). During this process, unreacted *i*-CO_2_ from the cathode gas stem was effectively utilized for anolyte acidification, achieving a state-of-the-art overall carbon utilization rate exceeding 97%. Acidification of the anolyte for sulfur extraction carries the risk of gaseous H_2_S evolution due to excessive acidity. In this study, however, this issue is mitigated by the slow and controlled acidification process achieved using a small amount of unreacted CO_2_ from the cathode outlet stream. Remarkably, leveraging the anolyte purification effect, the CO_2_ content in the product stream was reduced to below 3%. This enables continuous production of syngas with CO/H_2_ ratios ranging from 2:1 to 1:1, aligning with the upstream feedstock requirements for Fischer–Tropsch synthesis and synthesis of higher alcohols.

Preliminary analysis of electrolysis energy consumption and associated carbon emissions was conducted. As illustrated in Fig. 5c, conventional (bi)carbonate electrolyzers typically operate at 3.5–5.0 V cell voltage to achieve 100 mA cm^−2^ current density, with FE_CO_ ranging from 30%–80%. The specific energy consumption for CO production is 9–32 MWh t^−1^ (considering only the electrolyzer). In comparison, CO_2_ gas-fed electrolyzers exhibit lower cell voltages and enhanced product selectivity, reducing overall energy consumption to 6–19 MWh t^−1^. Through incorporation of SOR at the anode and optimization of BPM materials, our approach further decreases the energy requirement to ~6.5 MWh t^−1^ for bicarbonate electrolysis. When powered by solar energy with a carbon intensity of 0.05 t_CO2_ MWh^−1^, the optimized system achieves a net negative emission of –0.9 t_CO_2__ for per ton CO production, demonstrating a marked improvement over conventional (bi)carbonate electrolyzers (Fig. 5d). It should be noted that carbon emission analysis is critically dependent on the grid scenario. A detailed sensitivity analysis of emissions to the power generation mix is provided in Note S3 and Fig. S31. While the current study makes substantial progress in efficiency and energy reduction, we recognize opportunities for further improving long-term durability. Post-stability analysis revealed dark precipitates in the anolyte (Fig. S32), indicating SOR catalyst detachment from the porous electrode substrate. Furthermore, the CO/H_2_ ratio in the cathodic syngas gradually decreased from 1.9 to 1.3 during extended operation, suggesting degradation of the CO_2_RR catalyst. Future work should focus on enhancing electrode material stability to further extend operational lifetime. Moreover, advanced research systems have achieved a closed cycle enabling catholyte regeneration and reuse in (bi)carbonate electrolyzers through rational regulation of the rate matching between CO_2_ absorption and conversion, which significantly improves process feasibility and economic viability [17]. While current research focuses on the compatibility of paired anode and cathode reactions, the regeneration of the catholyte has not yet been realized. Future studies should therefore aim to accomplish a fully closed-loop system.

## CONCLUSION

In summary, we demonstrated a BPM-integrated paired electrolyzer for the co-upgrading of carbon capture solutions and sulfur-containing pollutants into syngas and sulfur. We revealed that the efficient water dissociation and mass transfer of BPMs are crucial for reconciling asymmetric pH requirements in paired electrolysis, thereby expanding the operational window to industrial grade current densities. We proposed that rational integration of downstream separation and purification processes with paired reactions presents a viable approach to streamline the electrolysis systems and enhances their operability. By combining numerical modeling, tailored high-performance BPMs, and advanced self-sustained system design, our electrolyzer achieved stable operation for over 300 h at a current density of 100 mA cm^−2^, with near-complete carbon utilization and high-purity syngas production (>97%). We identified that improving the physical and chemical stability of electrode materials remains the primary bottleneck for further extending paired electrolyzer lifetimes. Our study provides insights into the application of BPMs in various paired electrolysis systems, spanning from advanced material fabrication to integrated process optimization.

## METHODS

### Electrode preparation

For the preparation of Ag-coated hydrophilic carbon paper (Ag@carbon paper), 0.1 g of Ag nanoparticles and 0.5 g of BPSA ionomer solution (5.0 wt%) were dispersed in a mixed solvent comprising 0.4 mL of deionized water and 1.6 mL of isopropanol. The resulting catalyst ink was ultrasonicated to ensure homogeneity, spray-coated onto a hydrophilic carbon paper to form a GDE, and dried to achieve an Ag loading of ~2.0 mg cm^−2^. The commercially purchased Ag foam was directly used after immersion in nitric acid (3 mol L^−1^) for 30 s followed by thorough rinsing with deionized water.

The anode catalyst was grown on nickel foam via a two-step hydrothermal method following literature procedures [38]. Prior to synthesis, the nickel foam was sequentially cleaned with 3.0 mol L^−1^ HCl, ethanol, and deionized water. The pretreated substrate was then immersed in an aqueous solution containing 0.58 g Co(NO_3_)_2_•6H_2_O (2 mmol), 0.48 g urea (8 mmol), 0.11 g NH_4_F (3 mmol), and 40 mL deionized water. The hydrothermal reaction was conducted in a Teflon-lined autoclave, with the first step performed at 120°C for 3 h. After cooling to room temperature, the resulting precursor (Co(OH)_2_@nickel foam) was collected, washed thoroughly with deionized water, and dried. The resulting precursor was subsequently immersed in 0.3 mol L^−1^ Na_2_S•9H_2_O aqueous solution and reacted at 120°C for 3 h in the autoclave. After cooling to room temperature, the electrode (Co_3_S_4_@nickel foam) was retrieved and stored in deionized water.

### Ionomer synthesis

Both the anion and cation exchange ionomer (AEI and CEI) were synthesized from the same polymeric precursor, which was prepared via superacid-catalyzed polymerization following previously reported methods (see Supplementary Information for details) [49].

### Fabrication of bipolar membranes

The bipolar membranes were prepared according to our previously developed protocol. Briefly, a 5.0 wt% AEI solution in dimethyl sulfoxide (DMSO) was prepared and cast onto a glass substrate. The substrate was then dried at 80°C for 5 h on a hotplate to ensure complete solvent evaporation. Subsequently, a SnO_2_ nanoparticles dispersion (2.0 mg mL^−1^ in deionized water) was uniformly spray-coated onto the resulting anion exchange membrane (AEM) surface using ultrasonic spraying, with the water dissociation catalyst loading controlled at ~0.08 mg cm^−2^. For the subsequent layer, a 3.0 wt% CEI solution in DMSO/methanol (1:2, w/w) was spray-coated onto the catalyst layer, while elevating the substrate temperature to 90°C to facilitate rapid solvent evaporation. The membrane thickness could be precisely tuned by controlling the amount of ionomer solution during each step.

### Electrolyzer measurements

In the two-electrode flow cell configurations (Fig. S29), the cathode flow field plate was filled with 3 mol L^−1^ KHCO_3_ containing 0.02 mol L^−1^ ethylene diamine tetraacetic acid (EDTA) for preventing electrolyte impurities from electrodeposition [53], while the anode was filled with either 1 mol L^−1^ KOH (for OER) or a mixed solution of 1 mol L^−1^ KOH and 1 mol L^−1^ Na_2_S (for SOR) as the respective electrolytes. Both electrolytes were circulated using peristaltic pumps at a flow rate of 10 mL min^−1^, with the cathode and anode titanium flow-field plates (with an effective area of 1 cm^2^) separated by a membrane electrode assembly (MEA). The MEA consisted of a prefabricated Ag-based GDE cathode and a Co_3_S_4_@nickel foam anode (geometric area: 1 × 1 cm^2^), separated by either a PEM (Nafion 117, ~180 µm) or BPM (commercial Fumasep FBM-PK and tailor-made BPSn-BM with thicknesses of ~130 and ~50 µm, respectively). Polytetrafluoroethylene gaskets (with a thickness of 200 µm) were employed to seal the gaps between electrodes and flow field plates. The electrolyzer was powered by a CT3002N Land testing system (Land Electronics Co., Ltd, Wuhan). All electrolysis measurements were performed at room temperature. Initial stabilization was conducted at a low current density of 20 mA cm^−2^ for 2 h, followed by systematic recording of cell voltages (up to 5.0 V) at varied current densities to construct polarization curves. The self-sustained configuration is designed as follows: the cathodic gas-liquid mixture is separated via a dual-inlet buffer vessel, after which the gas stream is directed through a scrubbing bottle equipped with a gas sparger at its outlet for efficient gas-liquid extraction using the anolyte.

### Product analysis

The gaseous products were quantified by a gas flow meter and analyzed by an Agilent 8860 gas chromatographer (Agilent Technologies Inc., USA) equipped with a thermal conductivity detector and a flame ionization detector. The Faradaic efficiency (FE) of the gaseous product was calculated as follows:


(1) \begin{eqnarray*}
{\mathrm{F}}{{\mathrm{E}}}_k{\mathrm{\ = \ }}\frac{{{z}_k{\mathrm{\ \times \ }}F\ {\mathrm{ \times \ }}{\lambda }_k{\mathrm{\ \times }}\ {F}_m}}{I}{\mathrm{\ \times \ 100\% }},
\end{eqnarray*}

where *I* is the applied current in A; *z_k_* is the number of electrons transferred to form gaseous product *k* (*z_k_* = 2 both for CO and H_2_); *F* is Faraday’s constant (96 485 C mol^−1^); *λ_k_* is the molar fraction of *k; F_m_* represents the molar flow rate of the gas outlet stream in mol s^−1^, which is calculated as follows:


(2) \begin{eqnarray*}
{F}_m{\mathrm{\ = \ }}\frac{{p\ {\mathrm{ \times \ }}{F}_v}}{{R\ {\mathrm{ \times \ }}T}},
\end{eqnarray*}

where *p* is the atmospheric pressure (1.013 × 10^5^ Pa); *F_v_* is the volume flow rate measured by a gas flowmeter in m^3^ s^−1^; *R* is the ideal gas constant (8.314 m^3^ Pa mol^−1^ K^−1^); *T* is the temperature in K.

The CO_2_ utilization efficiency was calculated as follows:


(3) \begin{eqnarray*}
&&{\mathrm{CO_2\ utilization\ efficiency }}\nonumber\\
&&= \frac{{{\mathrm{[CO]\ }}}}{{{\mathrm{[CO]\ + \ [C}}{{\mathrm{O}}}_{\mathrm{2}}{\mathrm{]}}}}{\mathrm{\ \times \ 100\% }},
\end{eqnarray*}

where [CO] and [CO_2_] represent the concentrations of CO and CO_2_ in gaseous products, respectively.

The S^2−^ concentration in the anolyte was analyzed by the UV spectrophotometric method. Prior to the anolyte analysis, a calibration curve was established by measuring the absorption peak intensity of a series of standard solutions using UV-Vis spectroscopy at a wavelength of λ = 230 nm. The anolyte was periodically sampled, diluted 5000-fold, and subjected to UV-Vis analysis. The S^2−^ concentration was then determined by correlating the measured absorbance with the calibration curve. The S^2−^ conversion rate was calculated as follows:


(4) \begin{eqnarray*}
{\mathrm{Conversion\ rate}}\ {\mathrm{ = }}\ \frac{{{C}_{\mathrm{0}}{\mathrm{-}}{C}_t\ }}{{{C}_{\mathrm{0}}}}\ {\mathrm{ \times }}\ {\mathrm{100\% }},
\end{eqnarray*}

where *C*_0_ and *C_t_* represent the S^2−^ concentration in the anolyte at initial state and after *t* hours, respectively.

## Supplementary Material

nwaf504_Supplemental_Files

## References

[1] De Luna P, Hahn C, Higgins D et al. What would it take for renewably powered electrosynthesis to displace petrochemical processes? Science 2019; 364: eaav3506. 10.1126/science.aav3506 31023896

[2] Shin H, Hansen KU, Jiao F. Techno-economic assessment of low-temperature carbon dioxide electrolysis. Nat Sustain 2021; 4: 911–9. 10.1038/s41893-021-00739-x

[3] Lees EW, Mowbray BAW, Parlane FGL et al. Gas diffusion electrodes and membranes for CO_2_ reduction electrolysers. Nat Rev Mater 2021; 7: 55–64. 10.1038/s41578-021-00356-2

[4] O’Brien CP, Miao RK, Shayesteh Zeraati A et al. CO_2_ electrolyzers. Chem Rev 2024; 124: 3648–93. 10.1021/acs.chemrev.3c00206 38518224

[5] Endrődi B, Samu A, Kecsenovity E et al. Operando cathode activation with alkali metal cations for high current density operation of water-fed zero-gap carbon dioxide electrolysers. Nat Energy 2021; 6: 439–48. 10.1038/s41560-021-00813-w 33898057 PMC7610664

[6] Hao S, Elgazzar A, Zhang S-K et al. Acid-humidified CO_2_ gas input for stable electrochemical CO_2_ reduction reaction. Science 2025; 388: eadr3834. 10.1126/science.adr3834 40504913

[7] Rabinowitz JA, Kanan MW. The future of low-temperature carbon dioxide electrolysis depends on solving one basic problem. Nat Commun 2020; 11: 5231. 10.1038/s41467-020-19135-8 33067444 PMC7567821

[8] Lees EW, Bui JC, Romiluyi O et al. Exploring CO_2_ reduction and crossover in membrane electrode assemblies. Nat Chem Eng 2024; 1: 340–53. 10.1038/s44286-024-00062-0

[9] Salvatore DA, Gabardo CM, Reyes A et al. Designing anion exchange membranes for CO_2_ electrolysers. Nat Energy 2021; 6: 339–48. 10.1038/s41560-020-00761-x

[10] Zhu P, Wang H. High-purity and high-concentration liquid fuels through CO_2_ electroreduction. Nat Catal 2021; 4: 943–51. 10.1038/s41929-021-00694-y

[11] Alerte T, Edwards JP, Gabardo CM et al. Downstream of the CO_2_ electrolyzer: assessing the energy intensity of product separation. ACS Energy Lett 2021; 6: 4405–12. 10.1021/acsenergylett.1c02263

[12] Namdari M, Kim Y, Pimlott DJD et al. Reactive carbon capture using electrochemical reactors. Chem Soc Rev 2025; 54: 590–600. 10.1039/D4CS00834K 39635721

[13] Wang J, Zhu P, Qin H et al. Electrochemical reactors for the utilization of liquid-phase carbon species. Energy Environ Sci 2025; 18: 6438–55. 10.1039/D5EE01448D

[14] Li T, Lees EW, Goldman M et al. Electrolytic conversion of bicarbonate into CO in a flow cell. Joule 2019; 3: 1487–97. 10.1016/j.joule.2019.05.021

[15] Song H, Fernández CA, Choi H et al. Integrated carbon capture and CO production from bicarbonates through bipolar membrane electrolysis. Energy Environ Sci 2024; 17: 3570–9. 10.1039/D4EE00048J

[16] Venkataraman A, Song H, Brandão VD et al. Process and techno-economic analyses of ethylene production by electrochemical reduction of aqueous alkaline carbonates. Nat Chem Eng 2024; 1: 710–23. 10.1038/s44286-024-00137-y

[17] Kim Y, Lees EW, Donde C et al. Integrated CO_2_ capture and conversion to form syngas. Joule 2024; 8: 3106–25. 10.1016/j.joule.2024.10.010

[18] Li YC, Lee G, Yuan T et al. CO_2_ electroreduction from carbonate electrolyte. ACS Energy Lett 2019; 4: 1427–31. 10.1021/acsenergylett.9b00975

[19] Obasanjo CA, Gao G, Crane J et al. High-rate and selective conversion of CO_2_ from aqueous solutions to hydrocarbons. Nat Commun 2023; 14: 3176. 10.1038/s41467-023-38963-y 37264000 PMC10235047

[20] Xing K, Wang M, Pan B et al. Efficient bicarbonate electrolysis to formate enabled via ionomer surface modification in cation exchange membrane electrolyzers. Angew Chem Int Ed 2025; 64: e202504835. 10.1002/anie.202504835 40356034

[21] Ye F, Zhang S, Cheng Q et al. The role of oxygen-vacancy in bifunctional indium oxyhydroxide catalysts for electrochemical coupling of biomass valorization with CO_2_ conversion. Nat Commun 2023; 14: 2040. 10.1038/s41467-023-37679-3 37041142 PMC10090200

[22] Liu H, Shin H, Li XY et al. Hierarchically porous carbon supports enable efficient syngas production in electrified reactive capture. Energy Environ Sci 2025; 18: 6628–40. 10.1039/D5EE00094G

[23] Na J, Seo B, Kim J et al. General technoeconomic analysis for electrochemical coproduction coupling carbon dioxide reduction with organic oxidation. Nat Commun 2019; 10: 5193. 10.1038/s41467-019-12744-y 31729357 PMC6858374

[24] Gao W, Wang C, Wen W et al. Electrochemical hydrogen production coupling with the upgrading of organic and inorganic chemicals. Adv Mater 2025; 37: 2503198. 10.1002/adma.202503198 40395197

[25] Yu Z, Liu L. Recent advances in hybrid seawater electrolysis for hydrogen production. Adv Mater 2024; 36: 2308647. 10.1002/adma.202308647 38143285

[26] Jiang X, Ke L, Zhao K et al. Integrating hydrogen utilization in CO_2_ electrolysis with reduced energy loss. Nat Commun 2024; 15: 1427. 10.1038/s41467-024-45787-x 38365776 PMC10873292

[27] Verma S, Lu S, Kenis PJA. Co-electrolysis of CO_2_ and glycerol as a pathway to carbon chemicals with improved technoeconomics due to low electricity consumption. Nat Energy 2019; 4: 466–74. 10.1038/s41560-019-0374-6

[28] Wang X, Li P, Tam J et al. Efficient CO and acrolein co-production via paired electrolysis. Nat Sustain 2024; 7: 931–7. 10.1038/s41893-024-01363-1

[29] Lu C, Shi P, Huang S et al. Heteroarchitectural gas diffusion layer promotes CO_2_ reduction coupled with biomass oxidation at ampere-level current density. Angew Chem Int Ed 2025; 64: e202423263. 10.1002/anie.202423263 39777826

[30] Mou H, Lu F, Zhuang Z et al. Glycerol electrooxidation over precision-synthesized gold nanocrystals with different surface facets. Precis Chem 2024; 2: 103–11. 10.1021/prechem.3c00105 38550915 PMC10966739

[31] Zhang L, Wang Z, Qiu J. Energy-saving hydrogen production by seawater electrolysis coupling sulfion degradation. Adv Mater 2022; 34: 2109321. 10.1002/adma.202109321 35150022

[32] He D, Yang P, Yang K et al. Long-lasting hybrid seawater electrolysis enabled by anodic mass transport intensification for energy-saving hydrogen production. Adv Funct Materials 2024; 34: 2407601. 10.1002/adfm.202407601

[33] Yang K, Zhang N, Yang J et al. Synergistic marriage of CO_2_ reduction and sulfide oxidation towards a sustainable co-electrolysis process. Appl Catal, B 2023; 332: 122718. 10.1016/j.apcatb.2023.122718

[34] Gong S, Han X, Li W et al. Paired electrolysis for efficient coproduction of CO and S_8_ with techno-economic analysis. Chem Eng J 2025; 507: 160286. 10.1016/j.cej.2025.160286

[35] Zhang M, Guan J, Tu Y et al. Highly efficient H_2_ production from H_2_S via a robust graphene-encapsulated metal catalyst. Energy Environ Sci 2020; 13: 119–26. 10.1039/C9EE03231B

[36] Yi L, Ji Y, Shao P et al. Scalable synthesis of tungsten disulfide nanosheets for alkali-acid electrocatalytic sulfion recycling and H_2_ generation. Angew Chem Int Ed 2021; 60: 21550–7. 10.1002/anie.202108992 34288331

[37] Li T, Wang B, Cao Y et al. Energy-saving hydrogen production by seawater electrolysis coupling tip-enhanced electric field promoted electrocatalytic sulfion oxidation. Nat Commun 2024; 15: 6173. 10.1038/s41467-024-49931-5 39039041 PMC11263359

[38] Xiao Z, Lu C, Wang J et al. Bifunctional Co_3_S_4_ nanowires for robust sulfion oxidation and hydrogen generation with low power consumption. Adv Funct Materials 2023; 33: 2212183. 10.1002/adfm.202212183

[39] Weng LC, Bell AT, Weber AZ. Towards membrane-electrode assembly systems for CO_2_ reduction: a modeling study. Energy Environ Sci 2019; 12: 1950–68. 10.1039/C9EE00909D

[40] Lees EW, Bui JC, Song D et al. Continuum model to define the chemistry and mass transfer in a bicarbonate electrolyzer. ACS Energy Lett 2022; 7: 834–42. 10.1021/acsenergylett.1c02522

[41] Pimlott DJD, Kim Y, Berlinguette CP. Reactive carbon capture enables CO_2_ electrolysis with liquid feedstocks. Acc Chem Res 2024; 57: 1007–18. 10.1021/acs.accounts.3c00571 38526508

[42] Lu X, Zhou C, Delima RS et al. Visualization of CO_2_ electrolysis using optical coherence tomography. Nat Chem 2024; 16: 979–87. 10.1038/s41557-024-01465-5 38429344

[43] Xie K, Miao RK, Ozden A et al. Bipolar membrane electrolyzers enable high single-pass CO_2_ electroreduction to multicarbon products. Nat Commun 2022; 13: 3609. 10.1038/s41467-022-31295-3 35750665 PMC9232613

[44] Yan Z, Zhu L, Li YC et al. The balance of electric field and interfacial catalysis in promoting water dissociation in bipolar membranes. Energy Environ Sci 2018; 11: 2235–45. 10.1039/C8EE01192C

[45] Oener SZ, Foster MJ, Boettcher SW. Accelerating water dissociation in bipolar membranes and for electrocatalysis. Science 2020; 369: 1099–103. 10.1126/science.aaz1487 32616669

[46] Shen C, Wycisk R, Pintauro PN. High performance electrospun bipolar membrane with a 3D junction. Energy Environ Sci 2017; 10: 1435–42. 10.1039/C7EE00345E

[47] Kao YL, Chen L, Boettcher SW et al. Divergent synthesis of bipolar membranes combining strong interfacial adhesion and high-rate capability. ACS Energy Lett 2024; 9: 2953–9. 10.1021/acsenergylett.4c01178

[48] Shehzad MA, Yasmin A, Ge X et al. Shielded goethite catalyst that enables fast water dissociation in bipolar membranes. Nat Commun 2021; 12: 9. 10.1038/s41467-020-20131-1 33397931 PMC7782813

[49] Yu W, Zhang Z, Luo F et al. Tailoring high-performance bipolar membrane for durable pure water electrolysis. Nat Commun 2024; 15: 10220. 10.1038/s41467-024-54514-5 39587075 PMC11589674

[50] Zhang Z, Lees EW, Ren S et al. Conversion of reactive carbon solutions into CO at low voltage and high carbon efficiency. ACS Cent Sci 2022; 8: 749–55. 10.1021/acscentsci.2c00329 35756379 PMC9228564

[51] Teng X, Shi K, Chen L et al. Coupling electrochemical sulfion oxidation with CO_2_ Reduction over highly dispersed p-Bi nanosheets and CO_2_-assisted sulfur extraction. Angew Chem Int Ed 2024; 63: e202318585. 10.1002/anie.202318585 38108649

[52] Zhang Z, Lees EW, Habibzadeh F et al. Porous metal electrodes enable efficient electrolysis of carbon capture solutions. Energy Environ Sci 2022; 15: 705–13. 10.1039/D1EE02608A

[53] Wuttig A, Surendranath Y. Impurity ion complexation enhances carbon dioxide reduction catalysis. ACS Catal 2015; 5: 4479–84. 10.1021/acscatal.5b00808

