# Supplementary material for: Bipolar membrane electrolyzers for co-upgrading of CO2 capture solutions and sulfide contaminants to syngas and sulfur
Source: Natl Sci Rev. 2025 Nov 19;13(3):nwaf504. doi: 10.1093/nsr/nwaf504 (PMC12895916; doi:10.1093/nsr/nwaf504)
Supplement: nwaf504_Supplemental_Files [file nwaf504_supplemental_files.zip › 1590-Supplementary Information.pdf]

Supplementary Information

Bipolar membrane electrolyzers for co-upgrading of CO_2 capture solutions and sulfide contaminants to syngas and sulfur

Weisheng Yu[1,†], Fen Luo[1,†], Xian Liang[1], Xiaojiang Li[1], Wenfeng Li[1], Jingjing Tu[1], Luxin Xiong[1], Jihao Zhang[1], Liang Wu[1,]* & Tongwen Xu[1,]*

[1] State Key Laboratory of Precision and Intelligent Chemistry, Department of Applied Chemistry, School of Chemistry and Materials Science, University of Science and Technology of China, Hefei, 230026, China.

* Corresponding authors. Email: liangwu8@ustc.edu.cn (L. W.); twxu@ustc.edu.cn (T. X.)

[†] These authors contributed equally to this work.

Supplementary Methods

Materials

Silver (Ag) nanoparticles (99.99%, ~ 20 nm) were purchased from Zhongke Keyou Technology Co., Ltd (Beijing, China). Pt–Ru/C (HPR060, 60 wt% Pt-Ru on carbon black) was purchased from Shanghai Hesen Electric Co., Ltd (Shanghai, China). Nickel foam (0.5 mm, areal density of 280 g m^{-2}) and hydrophilic carbon paper (TGP-H-060SH, 0.2 mm) were purchased from Suzhou Sinero Technology Co., Ltd (Suzhou, China). Cobalt(II) nitrate hexahydrate ($Co(NO_3)_2•6H_2O$, 98%), ammonium fluoride (NH_4F, 97%), urea (99%), sodium sulfide nonahydrate ($Na_2S•9H_2O$, 99%), biphenyl (99%), 7-bromo-1,1,1-trifluoroheptan-2-one (98%), trifluoromethanesulfonic acid (TFSA, 99%), trimethylamine (2.0 mol L^{-1} in ethanol), potassium thioacetate (98%), m-chloroperoxybenzoic acid (85%), ethylenediaminetetraacetic acid (EDTA, 99.5%), and N,N-dimethylacetamide (DMAc, 99.8%, extra dry) were purchased from Energy Chemical Co., Ltd (Shanghai, China). Isopropanol (AR), ethanol (AR), dichloromethane (DCM, AR), methanol (AR), dimethyl sulfoxide (DMSO, AR), ethyl acetate (AR), hydrochloric acid (HCl, 36–38%), sodium chloride (NaCl, AR), potassium bicarbonate ($KHCO_3$, AR), and potassium hydroxide (KOH, AR) were purchased from Sinopharm Chemical Reagent Co.,Ltd (Shanghai, China). All materials were used as received without further purification.

Anion and cation exchange ionomer synthesis

Both the anion and cation exchange ionomer (AEI and CEI) were synthesized from the same polymeric precursor. In short, 4.62 g biphenyl (30 mmol) and 8.15 g 7-bromo-1,1,1-trifluoroheptan-2-one (33 mmol) were dissolved in 20 mL DCM, followed by dropwise addition of 20 mL TFSA under ice-bath cooling. The reaction mixture was then stirred at room temperature for 12 h. The resulting highly viscous gel-like product was precipitated into methanol, washed thoroughly with methanol, and collected as white fibers. The resulting bromoalkyl-tethered precursor (BPBr) was vacuum-dried prior to subsequent synthesis.

The synthesis of the AEI (BPQA) is briefly described as follows. 3.0 g of BPBr (7.8 mmol bromoalkyl groups) was suspended in 30 mL DMSO, followed by the addition of 10 mL trimethylamine solution (20 mmol). The mixture was stirred at 40 °C for 12 h, during which the

polymer precursor gradually transitioning from an insoluble suspension to a homogeneous solution. The product was then precipitated into ethyl acetate, thoroughly washed with ethyl acetate, and dried under vacuum to afford a light-colored fibrous.

Synthesis of the CEI (BPSA) via a two-step procedure described as follows. 3.0 g of BPBr was dissolved in 30 mL DMAc, followed by the addition of 1.0 g potassium thioacetate (8.6 mmol). The reaction mixture was stirred at 50 °C for 5 h. Upon completion, the product was precipitated and washed thoroughly with methanol, and dried under vacuum. The resulting white fibrous solid was redissolved in 60 mL DMAc and cooled in an ice bath. 4.4 g of m-chloroperoxybenzoic acid (21.8 mmol) was added dropwise to the solution, resulting in the formation of a highly viscous gel. The product was then precipitated in 1 mol L^{-1} NaCl solution, collected by filtration, and thoroughly washed with deionized water. After vacuum drying, the desired white solid product was obtained.

Preparation of Pt–Ru/C-coated Ni foam

Pt–Ru/C@Ni foam was prepared via a spray-coating method as the SOR electrode for comparison. Briefly, 0.1 g Pt–Ru/C and 0.5 g of BPQA ionomer solution (5.0 *wt*%) were dispersed in a mixed solvent comprising 0.4 mL of deionized water and 1.6 mL of isopropanol. The resulting catalyst ink was ultrasonicated to ensure homogeneity, spray-coated onto a h Ni foam to form a gas diffusion electrode (GDE), and dried to achieve a Pt–Ru loading of ~ 1.0 mg cm^{-2}.

Three-electrode electrochemical measurements

The electrochemical measurements for the oxygen evolution reaction (OER) and sulfion oxidation reaction (SOR) were performed using a standard three-electrode configuration on a CHI 760E electrochemical workstation. The as-prepared anode served as the working electrode (effective area: 0.5 × 0.5 cm^2), while a Hg/HgO electrode (in 1 mol L^{-1} KOH) and a Pt foil were employed as the reference and counter electrodes, respectively. Linear sweep voltammetry (LSV) for OER was conducted in O_2-saturated 1 mol L^{-1} KOH, while SOR measurements were carried out in 1 mol L^{-1} KOH containing 1 mol L^{-1} Na_2S. Both measurements utilized a scan rate of 5 mV s^{-1} at a room temperature. Prior to LSV curves collection, electrode activation was conducted at a faster

scan rate of 20 mV s^{-1}. The electrode potentials measured were converted to the value against RHE according to the equation:

$$E_{(vs.\ RHE)} = E_{(vs.\ RHE)} + 0.059 \times pH + 0.098\ \mathrm{V} \quad \text{Eq. 1}$$

Ionic conductivity measurements

The ionic conductivity (σ) of the monopolar membranes was measured using an electrochemical workstation (AutoLab Zahner Zennium E, Germany) following our established method [1]. The AEM and CEM were first cut into rectangular samples (1 × 4 cm) and converted to OH^- and H^+ forms by immersing in NaOH (1.0 M) and H_2SO_4 (0.5 M) solution for 24 h, respectively. Before the tests, the samples were thoroughly washed with pure water. The electrochemical impedance spectroscopy (EIS) measurements were performed under a galvanostatic mode with the AC current amplitude of 10 μA and a frequency range from1 MHz to 100 Hz at a given temperature. Ohmic resistance extracted from the EIS measurements was recorded as R. The σ is calculated as:

$$\sigma = \frac{L}{R \times W \times d} \quad \text{Eq. 2}$$

where L is the distance between two potential-sensing electrodes of the four-electrode setup (1 cm), W and d are the width and thickness of the samples at the corresponding temperature, respectively. Each sample batch was measured three times, and the average values were calculated.

BPM water dissociation electrochemical measurements

The BPM water dissociation electrochemical measurements, including current-voltage (I–V) and electrochemical impedance spectroscopy (EIS), were measured using a custom-made four-electrode setup. Before the measurements, the BPM samples were pretreated by immersing them in 0.5 mol L^{-1} Na_2SO_4 solution for 12 h to minimize the noise effect. As shown in **Fig. S22**, the as-prepared BPM was stuck in the middle of two symmetrical compartments with an effective area of 3.14 cm^2, both the two compartments filled with 0.5 mol L^{-1} Na_2SO_4 solution. Two Pt electrodes placed outboard act as working electrode and counter electrode. Two reference electrodes (Ag/AgCl) were placed inside the Luggin capillary, which contacts the surface of BPMs. All the electrochemical properties were measured under a reverse bias configuration; namely, the

CEL faces the cathode, and the AEL faces the anode.

To obtain the *I*–*V* curves, an Autolab workstation (PGSTAT 302 N, Metrohm, Netherlands) connected with Nova 2.1.2 software was conducted under a galvanodynamic setup with a current range from 0 to 320 mA and a rate of current change of 10 mA s^{-1}. All tests were conducted at room temperature, and experiments were cut off when scanning to 320 mA. EIS experiments were performed at varying current densities (10, 20, 50, 100 mA cm^{-2}) in galvanostatic mode, with a 10% amplitude AC bias and spanning a frequency range from 100 kHz to 0.1 Hz. The obtained Nyquist plots were fitted using a Z-view software based on an equivalent circuit (**Fig. S23**), allowing to describe the ohmic resistance (R_{Ω}) and water dissociation reaction resistance (R_{WD}).

Characterizations

Field-emission scanning electron microscopy (FE-SEM, Gemini SEM 500, Germany) and energy-dispersive X-ray spectroscope (EDS) mapping were performed to investigate the microstructure of the electrode materials and the interfacial morphology of the BPMs. The surface SEM images of the electrodes were directly collected under an accelerating voltage of 3.0 kV. EDS mapping images were collected under a relatively higher accelerating voltage of 8.0 kV. The cross-section BPM samples were prepared with a single-edge blade and then vertically attached to the sample holder using carbon tape. The samples were sputter-coated with a layer of gold (with 1 nm thickness) before the test.

X-ray diffraction (XRD) patterns were recorded in the 2θ range of 10–60° at room temperature using a Rigaku X-ray Diffractometer Model TTR-III (Tokyo, Japan). The Co-based catalysts were directly loaded on nickel foam for XRD analysis. Following acidification of the anodic SOR products, the resulting light-yellow powder was rigorously purified through sequential washing and drying, with XRD confirming its composition as elemental sulfur (S_8).

The ultraviolet absorption spectra were collected using a UV–vis spectrometer (TU-1901, Beijing Purkinje General Instrument Co., Ltd.). The calibration curve was obtained by measuring the UV–vis spectrum of a series of standard solutions with varying concentrations (0, 0.02, 0.04, and 0.08 mM).

Energy requirement and process emission analyses

The energy consumption of the electrolyzer is correlated with the voltage and Faraday efficiency (*FE*). The total electricity requirement (W, in kWh t^{-1}) of the electrolyzers to produce 1 ton of CO is calculated as follows:

$$W = \frac{z \times F \times U \times m}{\mathrm{FE} \times M \times 3600000} \quad \text{Eq. 3}$$

Where z is the number of electrons transferred to CO (z = 2). F is Faraday's constant (96485 C mol^{-1}). U is the cell voltage in V. m = 1 ton = 1000000 g. M is molar mass of CO (M = 28 g mol^{-1}).

Total process emissions (e, in t_{CO_2} t_{CO}^{-1}) of the electrolyzer consist of CO_2 emissions ($e_{emission}$) from electricity consumption and CO_2 capture during electrolysis ($e_{capture}$). The former depends on electricity requirement and grid carbon intensity (e_{grid}, 0.05 t_{CO_2} kWh^{-1} from solar power generation), while the latter remains constant for a given product (1.22 t_{CO_2} t_{CO}^{-1}).

$$e = e_{emission} - e_{capture} = W \times e_{grid} - e_{capture} \quad \text{Eq. 4}$$

Based on the above equations, the process is net positive emissions if the CO_2 emissions from its electricity consumption exceed the CO_2 captured during electrolysis; otherwise, it achieves net negative emissions.

Numerical methods

The one-dimensional (1D) electrolysis model was established using COMSOL Multiphysics version 6.3, which incorporates multiple domains including a 200 µm cathode porous transport layer, a 180 µm PEM (to match the Nafion 117 membrane) or a BPM with thicknesses of 40 and 80 µm , a 200 µm anode diffusion medium (**Fig. S12**). The charge density of both the anion and cation exchange layer (AEL and CEL) of the BPM is set to 0.5 mmol cm^{-3}. CEL was modeled as a porous media using liquid-phase diffusion coefficients as per Weng *et al*. and Lees *et al* [2,3].

To simulate *i*-CO_2 generation, homogeneous buffer reactions (Eqs. 5–9) were established and treated as a system of kinetic expressions.

$$H_2O\ (l) \leftrightharpoons H^+\ (aq) + OH^-\ (aq) \quad K_W = 10^{-14}\ M^2 \quad \text{Eq. 5}$$

$$CO_2\ (aq) + H_2O\ (l) \leftrightharpoons HCO_3^-\ (aq) + H^+\ (aq) \quad K_1 = 10^{-6.37}\ M \quad \text{Eq. 6}$$

$$HCO_3^-\ (aq) \leftrightharpoons CO_3^{2-}\ (aq) + H^+\ (aq) \quad K_2 = 10^{-10.32}\ M \quad \text{Eq. 7}$$

$$CO_2\ (aq) + OH^- \ (aq) \leftrightharpoons HCO_3^-\ (aq) \quad K_3 = \frac{K_1}{K_W} \quad \text{Eq. 8}$$

$$HCO_3^-\ (aq) + OH^-\ (aq) \leftrightharpoons H_2O\ (l) + CO_3^{2-}\ (aq) \quad K_3 = \frac{K_2}{K_W} \quad \text{Eq. 9}$$

The Poisson-Nernst-Planck equation was used to model the liquid-phase chemical species (HCO_3^-, OH^-, H^+, and CO_2) and the gas-phase was assumed to behave as an ideal mixture. Water dissociation at the BPM interface generates H^+ and OH^-, with the H^+ flux was defined using Faraday's Law (Eq. 10).

$$n_{H^+}|_{x=0} = t_{H^+} \frac{1}{n_{WD}F} \int_{x = L_{CEL}}^{x = L_{CEL} + L_{CL}} i_{total}\, dx \quad \text{Eq. 10}$$

where n_{WD} represents the number of electrons consumed per mole of H^+ produced. F is the Faraday's constant (94685 C mol^{-1}), t is the transfer number, L is the length (m).

For the cathodic reaction, the partial current densities for CO_2RR (Eq. 11) and HER (Eq. 12) are given by Butler-Volmer expressions which depend on reactant concentrations at the electrode interface (Eq. 13).

$$CO_2\ (aq) + H_2O\ (l) + 2e^- \rightarrow CO\ (g) + 2OH^-\ (aq) \quad \text{Eq. 11}$$

$$2H^+\ (aq) + 2e^- \rightarrow H_2\ (g) \quad \text{Eq. 12}$$

$$i = i_0\ (e^{\frac{(1-\alpha) F \eta}{R T}} - \frac{c}{c_0}\ e^{\frac{-\alpha F \eta}{R T}}) \quad \text{Eq. 13}$$

Where i_0 is the partial exchange current densities, α is the transfer coefficient, η is the overpotential, R is universal gas constant (8.314 J mol^{-1} K^{-1}), T is the temperature (298.15 K).

Supplementary Figures

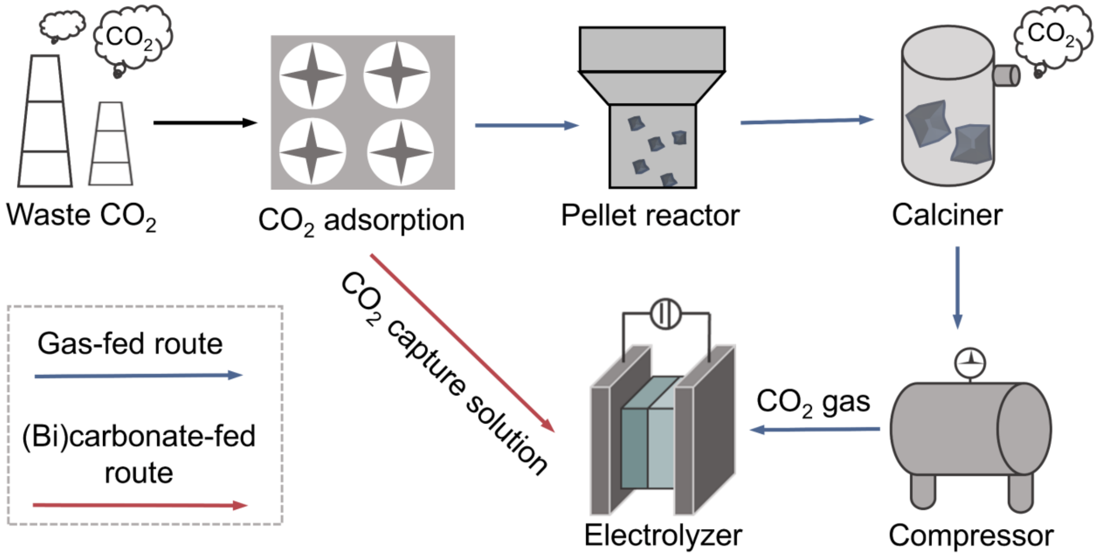

Figure S1. Route comparison of carbon capture solution *vs.* gaseous CO_2 electrolysis. Both electrolysis routes require CO_2 adsorption from dilute sources to generate capture liquid. However, the gaseous-fed approach necessitates energy-intensive intermediate steps (e.g., mineralization, calcination, and compression) to produce purified CO_2 for electrolysis (the top pathway). In contrast, liquid-fed electrolysis bypasses these processes, streamlining system operation and reducing overall energy demand (the bottom pathway).

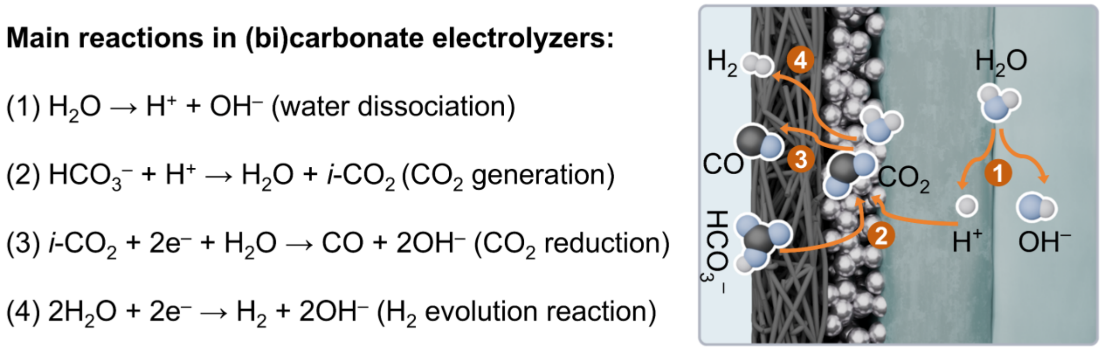

Figure S2. Reaction pathways in BPM bicarbonate (HCO_3^-) electrolyzer. (1) Water dissociation at the BPM interface generates H^+ that migrate to the cathode. (2) HCO_3^- react with these H^+ to form CO_2 *in situ*, (3) which is subsequently reduced to CO on the silver cathode. (4) Concurrently, a competitive hydrogen evolution reaction (HER) occurs at the cathode.

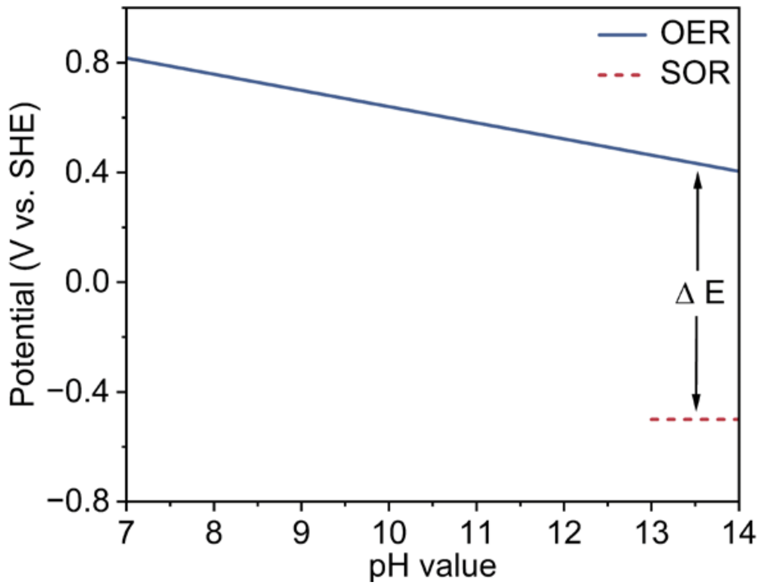

Figure S3. The Pourbaix diagram of OER and SOR under alkaline conditions, the SOR represented by a dashed line.

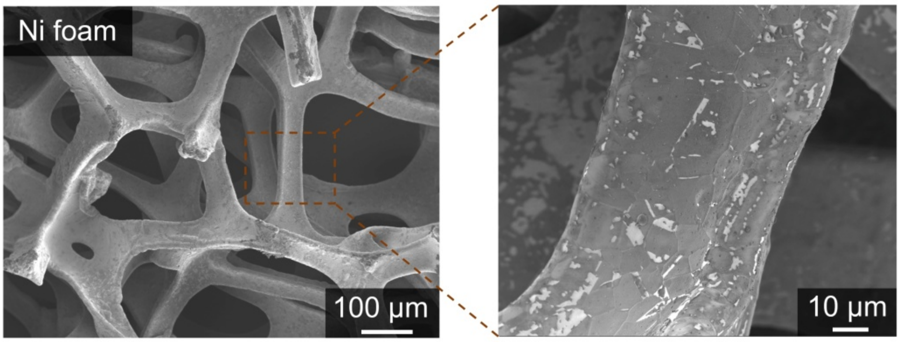

Figure S4. SEM images of the porous Ni foam. The magnifications are 120× (left) and 800× (right), respectively.

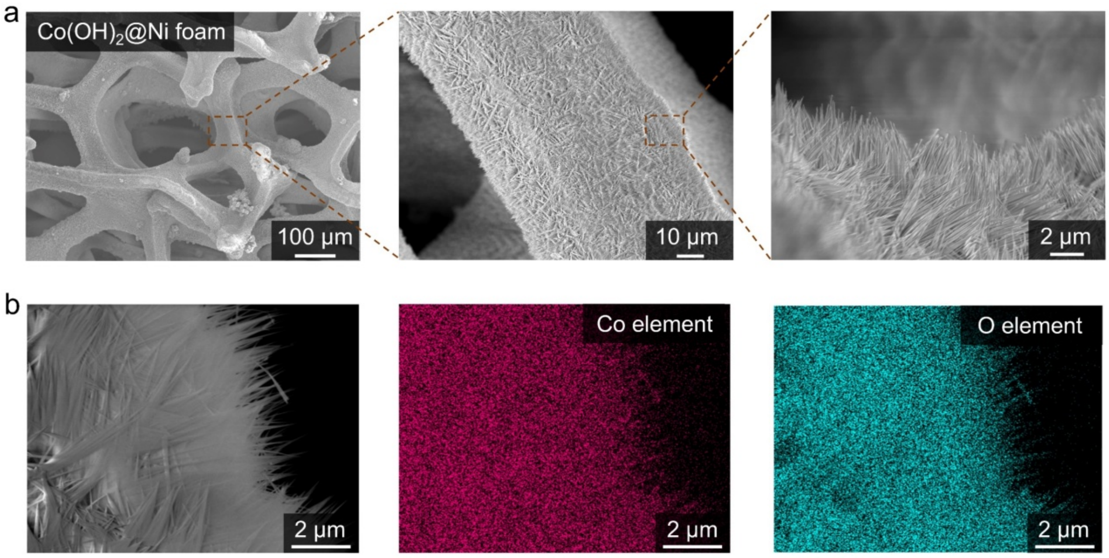

Figure S5. SEM and the corresponding EDX mapping images of the $Co(OH)_2$@Ni foam. (a) SEM images, the magnifications are 120× (left), 800× (middle), and 5000× (right) respectively. (b) EDX mapping images of Co and O elements for $Co(OH)_2$@Ni foam.

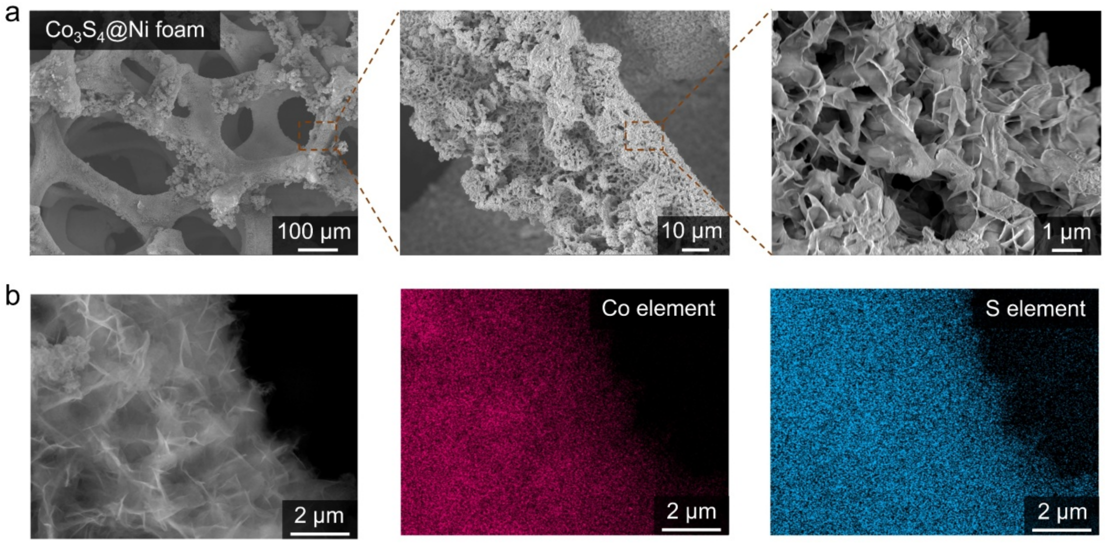

Figure S6. SEM and the corresponding EDX mapping images of the Co_3S_4@Ni foam. (a) SEM images, the magnifications are 120× (left), 800× (middle), and 10000× (right) respectively. (b) EDX mapping images of Co and S elements for Co_3S_4@Ni foam.

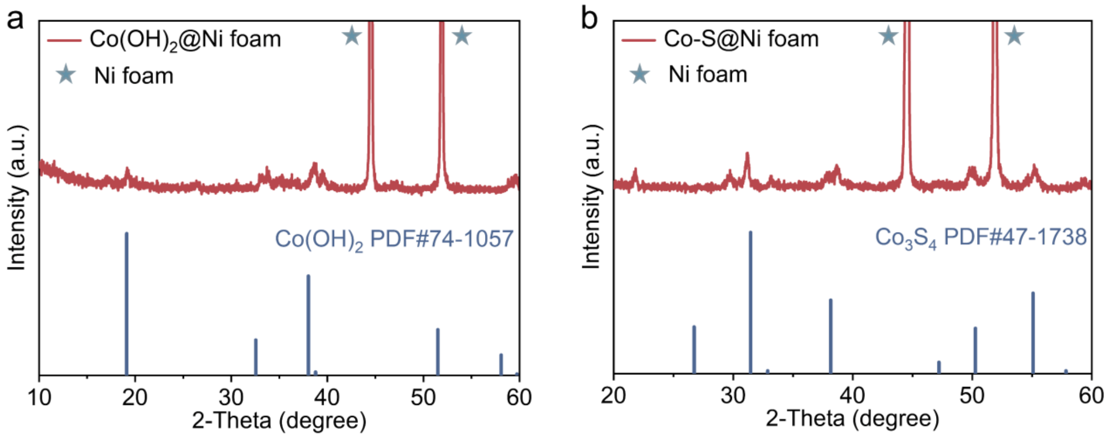

Figure S7. XRD patterns of (a) the $Co(OH)_2$@Ni foam, and (b) Co_3S_4@Ni foam. The characteristic Ni signals dominate the spectrum due to direct measurement on the Ni foam substrate.

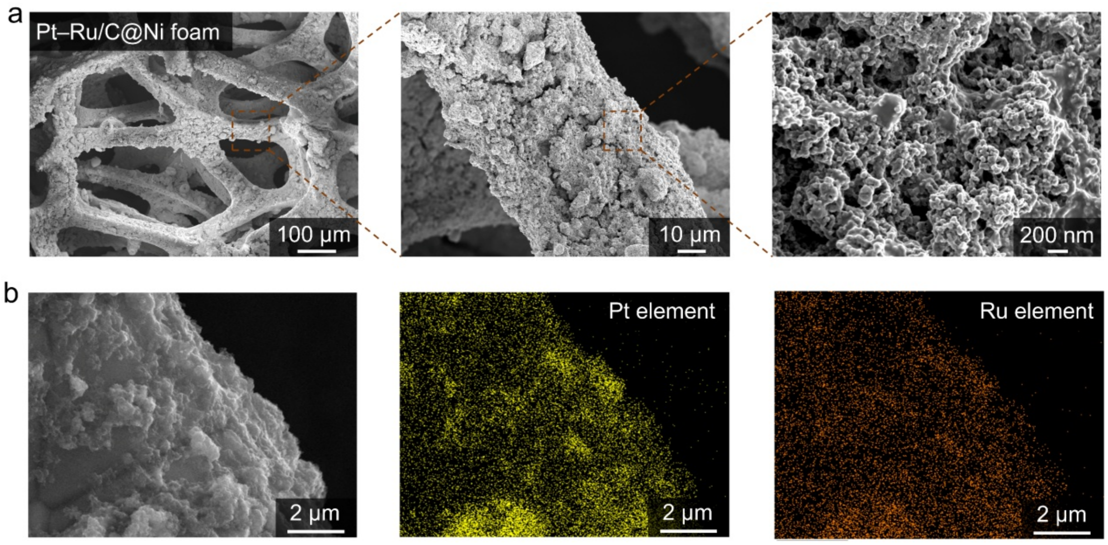

Figure S8. SEM and the corresponding EDX mapping images of the Pt–Ru/C@Ni foam. (a) SEM images, the magnifications are 120× (left), 800× (middle), and 30000× (right) respectively. (b) EDX mapping images of Pt and Ru elements for Co_3S_4@Ni foam.

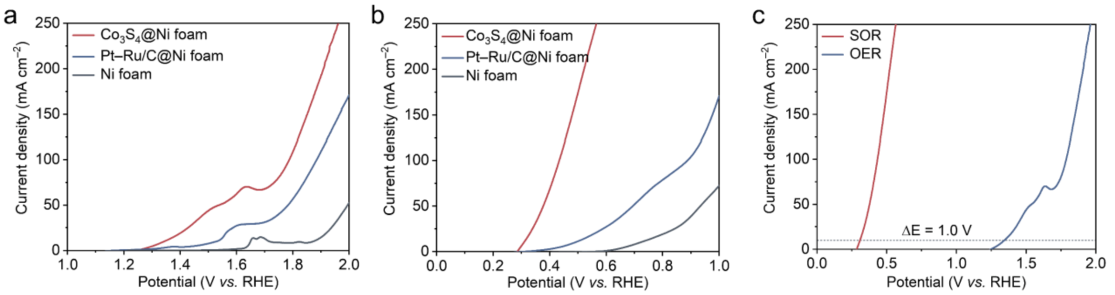

Figure S9. Electrochemical performance of Ni foam, Pt–Ru/C@Ni foam, and Co–S@Ni foam for OER and SOR. (a) OER LSV curves of Ni foam, Pt–Ru/C@Ni foam, and Co–S@Ni foam in 1.0 mol L^{-1} KOH. (b) SOR LSV curves of Ni foam, Pt–Ru/C@Ni foam, and Co–S@Ni foam in 1.0 mol L^{-1} KOH and 1.0 mol L^{-1} Na_2S. (c) Electrochemical activity comparison of Co–S catalyst for OER and SOR, showing a 1.0 V negative shift in onset potential (at current density of 10 mA cm^{-2}) for SOR relative to OER.

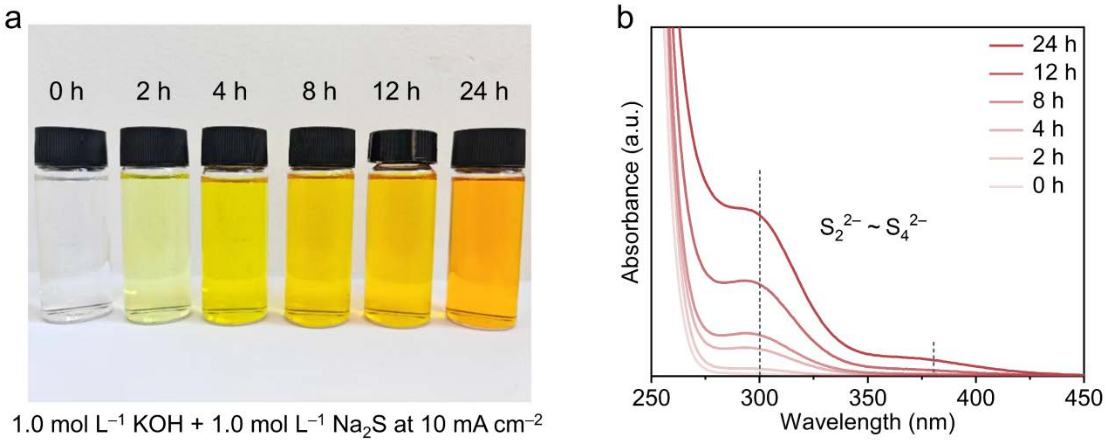

Figure S10. (a) Color evolution of the electrolyte with prolonged electrolysis time form 0 to 24 h at 10 mA cm^{-2}. (b) The UV–vis spectra of the anolyte diluted 500 times.

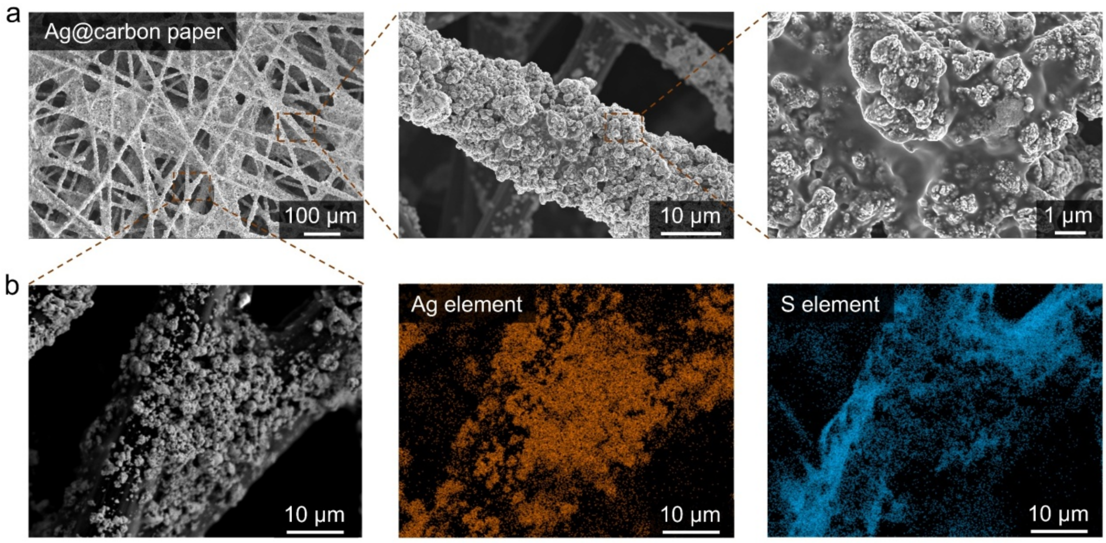

Figure S11. SEM and the corresponding EDX mapping images of the Ag nanoparticles@carbon paper. (a) SEM images, the magnifications are 120× (left), 2000× (middle), and 10000× (right) respectively. (b) EDX mapping images of Ag and S elements for the Ag nanoparticles and BPSA ionomer.

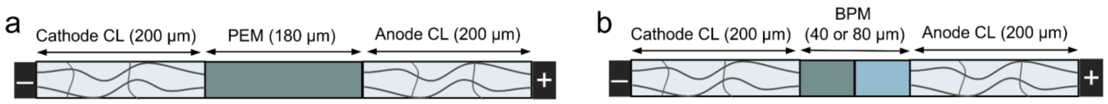

Figure S12. Schematic of modeling configurations of the (a) PEM-integrated, and (b) BPM-integrated electrolyzers. The PEM thickness was set to 180 μm to match commercial Nafion 117 membranes. Two thicknesses (40 and 80 μm) were evaluated for the bipolar membrane.

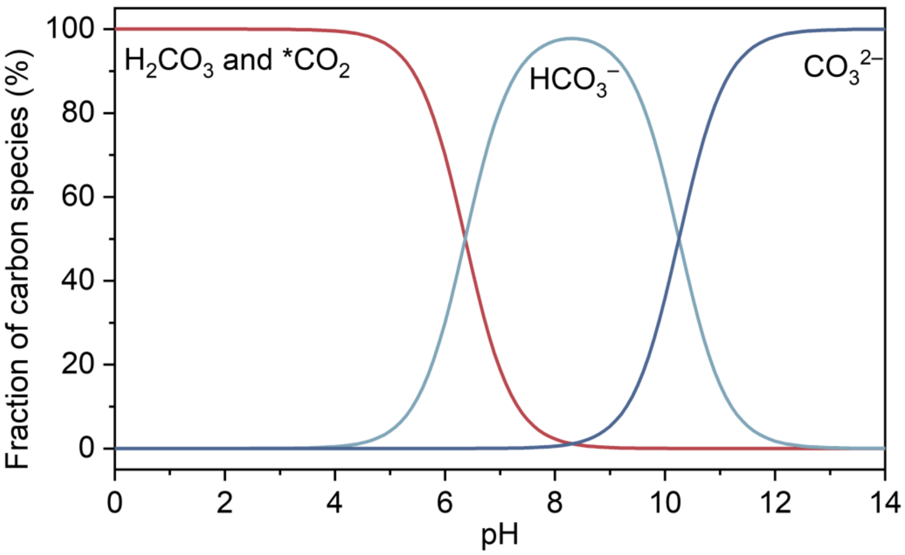

Figure S13. Relationship between carbon dioxide equilibrium and pH (with a temperature of 25 °C).

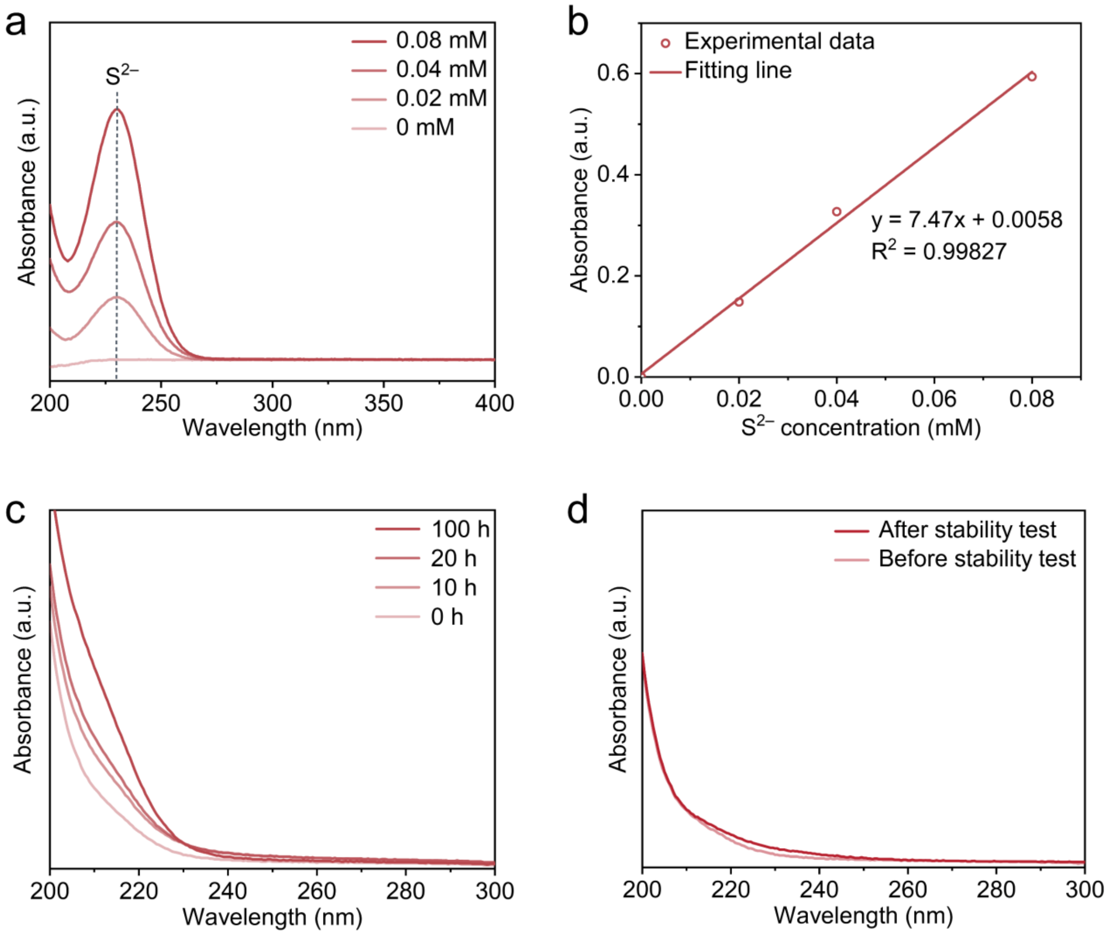

Figure S14. UV–vis spectra for S^{2-} detection. (a) The UV–vis spectra of a series of standard Na_2S solutions with varying concentrations (0, 0.02, 0.04, and 0.08 mM) and (b) the corresponding calibration curve. (c) The UV–vis spectra of the PEM electrolyzer catholyte at different times. The enhanced peak intensity at 225 nm indicates S^{2-} migration from the anolyte to the catholyte through the PEM. (d) The UV–vis spectra of the BPM electrolyzer catholyte before and after stability testing. Unlike PEM electrolyzers, the post-test catholyte showed no significant increase in S^{2-} concentration, demonstrating the superior species-blocking capability of BPMs.

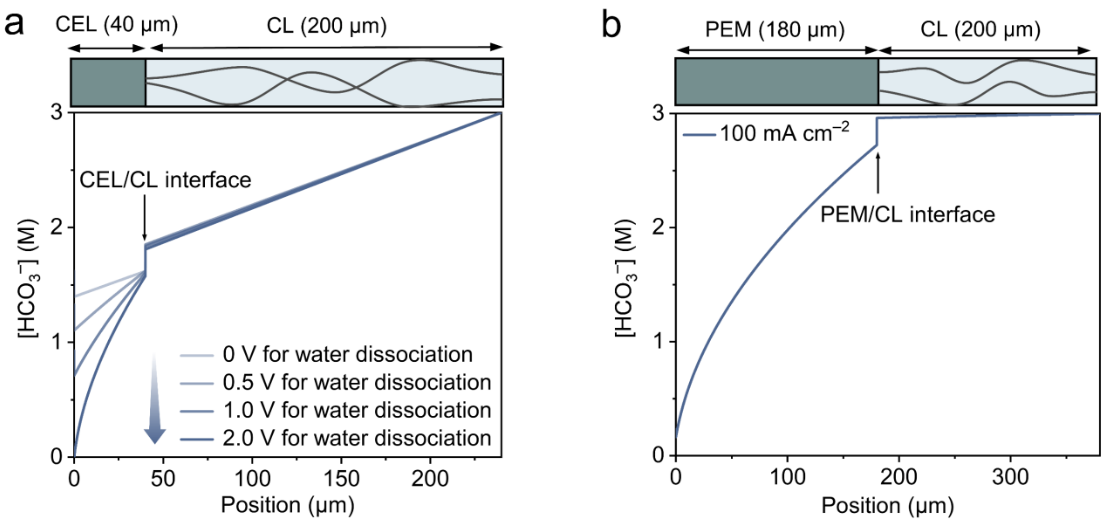

Figure S15. Modeled HCO_3^- concentration distribution within (a) the BPM electrolyzer at different water dissociation voltage, and (b) the PEM electrolyzer at current density of 100 mA cm^{-2}.

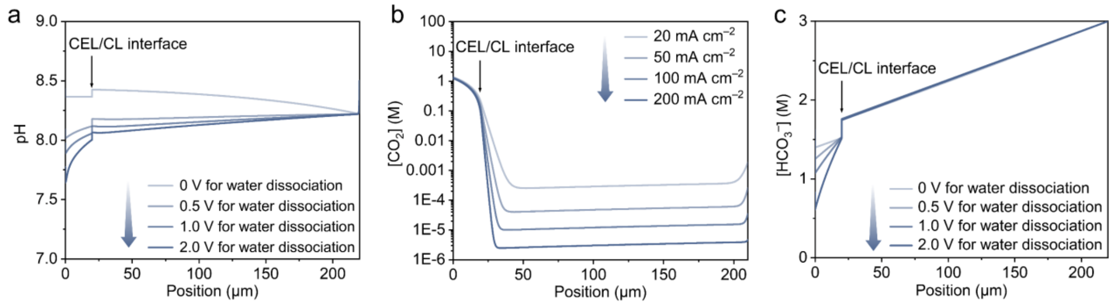

Figure S16. Modeled (a) pH, (b) CO_2 concentration, and (c) HCO_3^- concentration distribution within the 20 μm BPM electrolyzer.

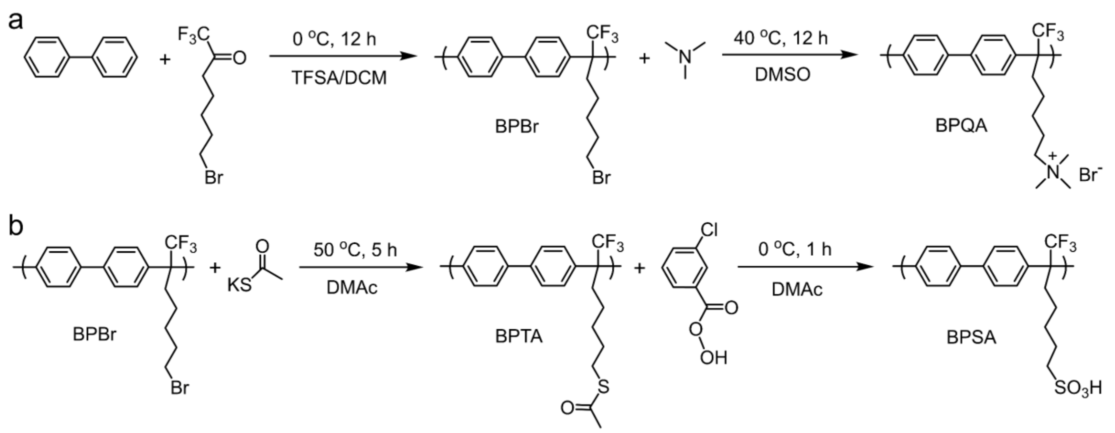

Figure S17. Synthesis routes of the anion- and cation exchange ionomers. (a) Synthetic pathways to bromoalkyl-tethered precursor polymer (BPBr) and quaternary ammonium-tethered anion exchange ionomer (BPQA). (b) Synthetic pathways to sulfonate-tethered proton exchange ionomer (BPSA).

Supplementary Note 1: Fabrication of BPMs.

We selected BPQA and BPSA as the AEL and CEL for the BPM owing to their excellent physicochemical properties, including superior mechanical strength, high ionic conductivity, and chemical stability. Additionally, the identical polymer backbones of the AEL and CEL ensure good compatibility between them, thereby enhancing interfacial strength. These contents have been validated in our previous work [1], and were featured as a research highlight in *Nature Energy* under the title 'Compatible Layers Bring Benefits' [4]. Notably, the fabrication of the BPSn-BM requires distinct processing methods for the two layers. For the bottom AEL, a casting method was employed: the AEI (BPQA) was dissolved in DMSO to form a homogeneous solution, which was then cast onto a glass plate and dried at 80 °C for 5 h. For the top CEL, a low-boiling-point DMSO/acetonitrile mixed solvent was used to dissolve the CEI, ensuring rapid drying during spray coating to prevent dissolution of the underlying AEL and damage to the bipolar interface. As shown in **Fig. 4a**, following this strategy we successfully fabricated a BPM with an area of 450 cm^2 (15 × 30 cm).

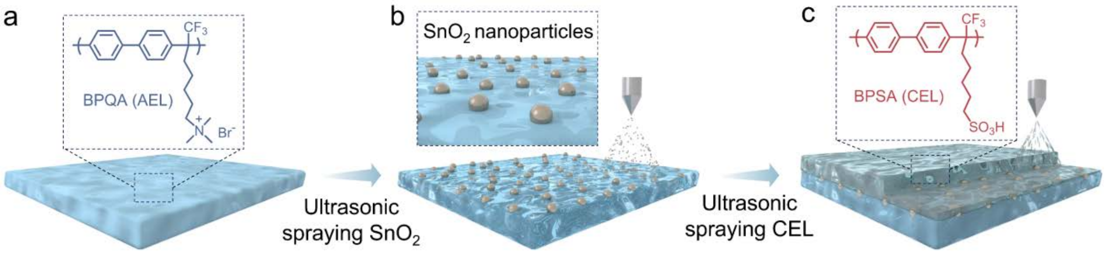

Figure S18. Fabrication of the BPSn-BM. The fabrication of a BPSn-BM involves three steps. (a) Casting a BPQA solution onto a glass plate, followed by solvent evaporation via heating to form the AEL; (b) spray-coating a SnO_2 suspension onto the AEL to create the water dissociation catalytic layer; and (c) spray-coating a BPSA solution onto the catalytic layer, followed by drying to obtain the final BPM.

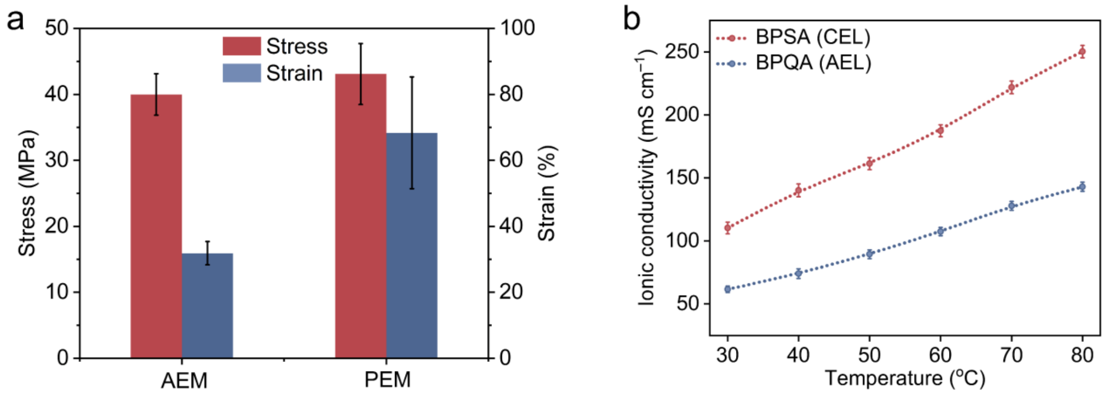

Figure S19. Fundamental properties of the monopolar membranes. (a) Mechanical properties of the monopolar membrane. (b) Temperature-dependent (from 30 to 80 °C) ionic conductivity of the monopolar membranes.

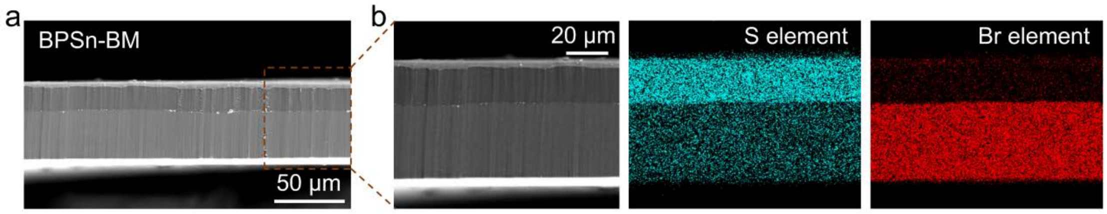

Figure S20. Cross-sectional SEM and the corresponding EDX mapping images of the BPSn-BM. (a) SEM images, the magnifications is 500×. (b) EDX mapping images of S and Br elements for the CEL and AEL, respectively.

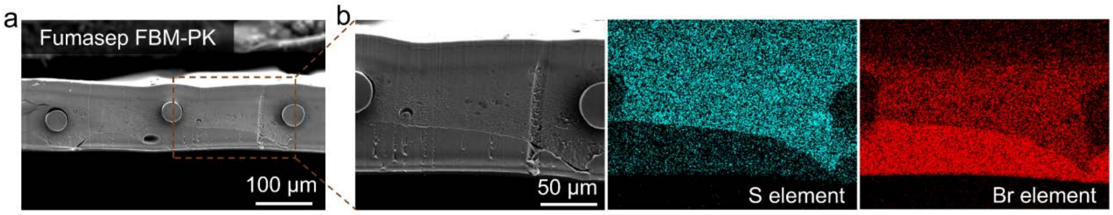

Figure S21. Cross-sectional SEM and the corresponding EDX mapping images of the commercial FBM-PK. (a) SEM images, the magnifications is 200×. (b) EDX mapping images of S and Br elements for the CEL and AEL, respectively.

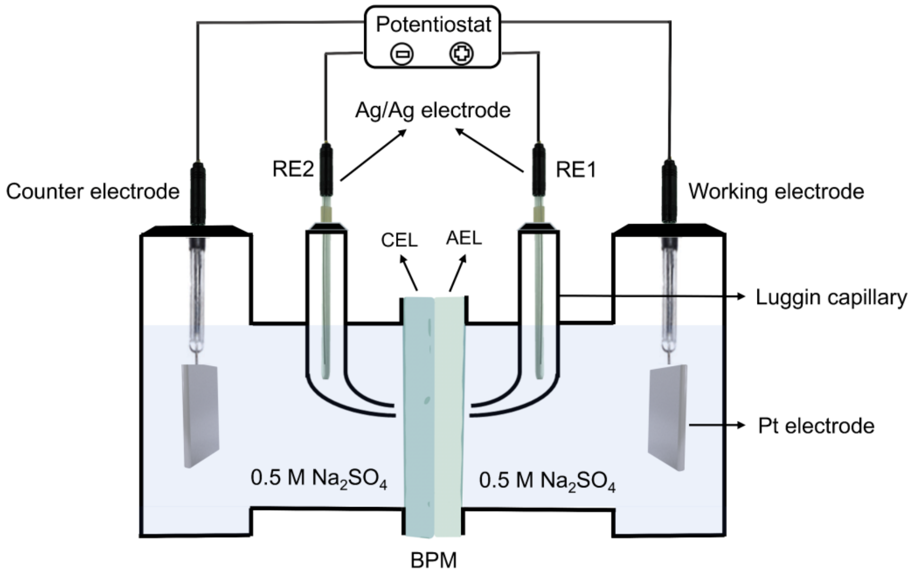

Figure S22. Schematic illumination of the custom-made four-electrode setup for BPM water dissociation electrochemical performance measurements. During the tests, the BPMs were stuck in the middle of two symmetrical compartments, which filled with 0.5 mol L^{-1} Na_2SO_4. Two Pt electrodes placed outboard act as working electrode and counter electrode. Two reference electrodes (Ag/AgCl) were respectively placed inside the Luggin capillary that contacts the surface of BPMs.

Supplementary Note 2: Water dissociation performance of the BPMs.

Under reverse bias (where the AEL and CEL face the anode and cathode, respectively), water molecules at the bipolar interface rapidly dissociate into H^+ and OH^-, facilitated by the enhanced electric field and catalytic effects [5,6]. EIS is normally performed to probe the water dissociation within BPMs, which differentiates the various components of the BPM as electrical features that can be derived from impedance responses upon a varying frequency [7]. In this way, the finite conductivity of the membrane layers is measured as an ohmic resistor (R_Ω), and the water dissociation reaction resistance is measured as R_{WD}.

We performed the EIS measurements using the four-electrode setup illustrated in **Fig. S22**, the resulting Nyquist plots for the FBM-PK and the BPSn-BM membranes are presented in **Figs. S23a and S23b**. R_Ω can be derived from the Nyquist plots by measuring the lengths between the origin and the start of the first semicircle, while the R_{WD} corresponds to the diameter of the semicircular arc. **Fig. 4b** compares the R_Ω and R_{WD} between FBM-PK and BPSn-BM membranes, revealing that the BPSn-BM demonstrates superior mass transport and water dissociation kinetics. At a current density of 100 mA cm^{-2}, for instance, the BPSn-BM exhibit R_Ω and R_{WD} of 0.13 and 0.02 Ω cm^2, respectively, significantly lower than the corresponding values of 0.59 and 0.66 Ω cm^2 for commercial BPSn-BM membrane.

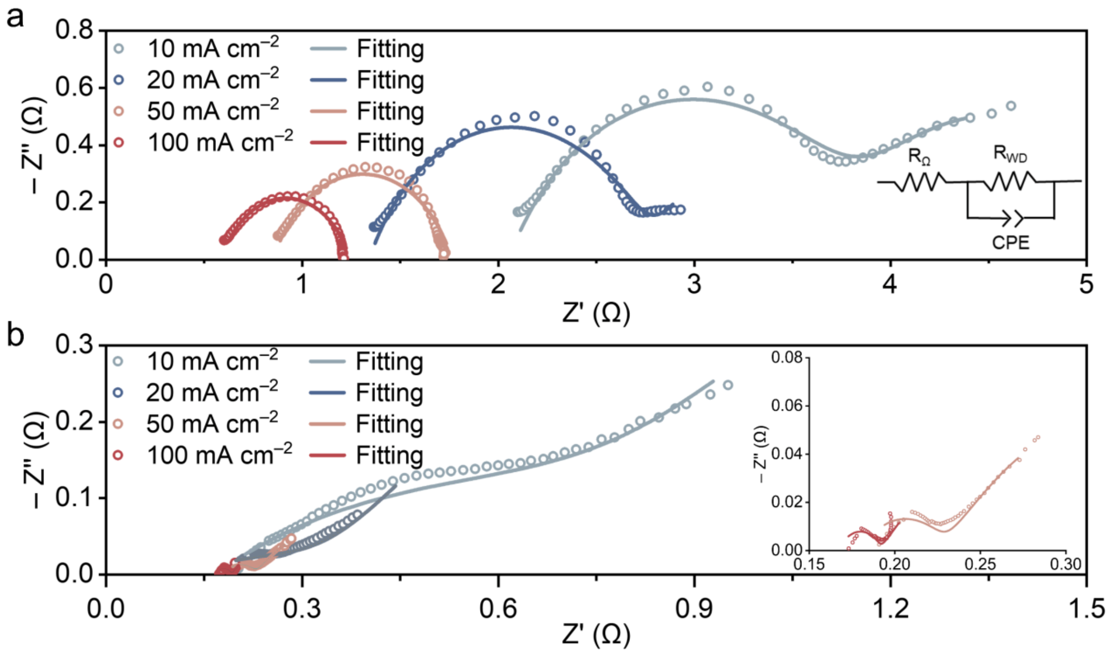

Figure S23. The EIS Nyquist plots and corresponding electrical equivalent circuit fitting curves of the (a) commercial FBM-PK, and (b) BPSn-BM at different current densities. Inset shows the equivalent circuit model, which is composed of a resistor (R_{Ω}), a block containing a resistor (R_{WD}) and a constant phase element (CPE) connected in parallel.

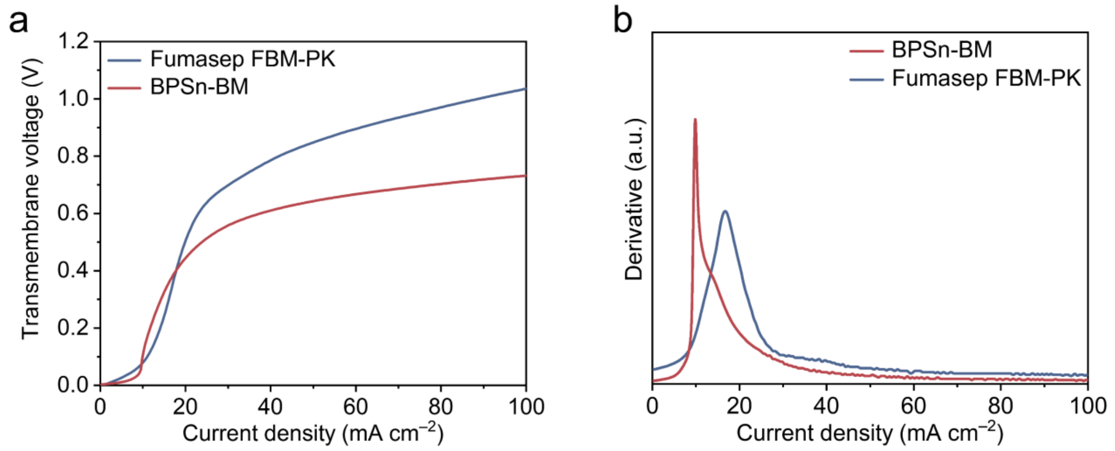

Figure S24. Water dissociation performance of the BPMs. (a) The current–voltage curves and (b) corresponding derivative curves of the TPSn-BM and FBM-PK at current densities of 0–100 mA cm^{-2}.

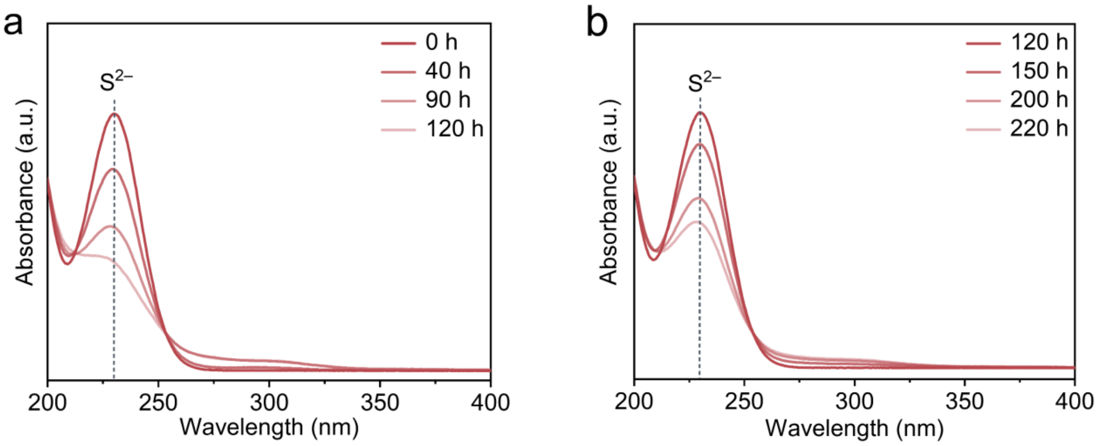

Figure S25. The UV–vis spectra of the anolyte diluted 5000-fold at different time intervals. (a) and (b) represent measurements before and after anolyte renewal, respectively.

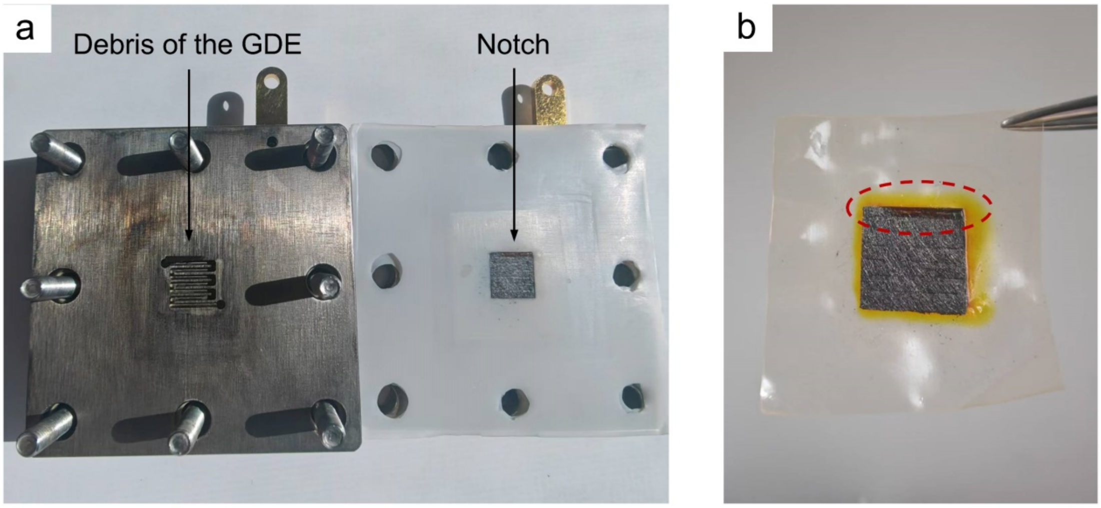

Figure S26. Post-stability-test autopsy digital photos of the membrane electrode assembly (MEA). Debris from the gas diffusion electrode (GDE) at the cathode flow channel outlet indicates carbon paper fracture under combined gas/liquid flow erosion.

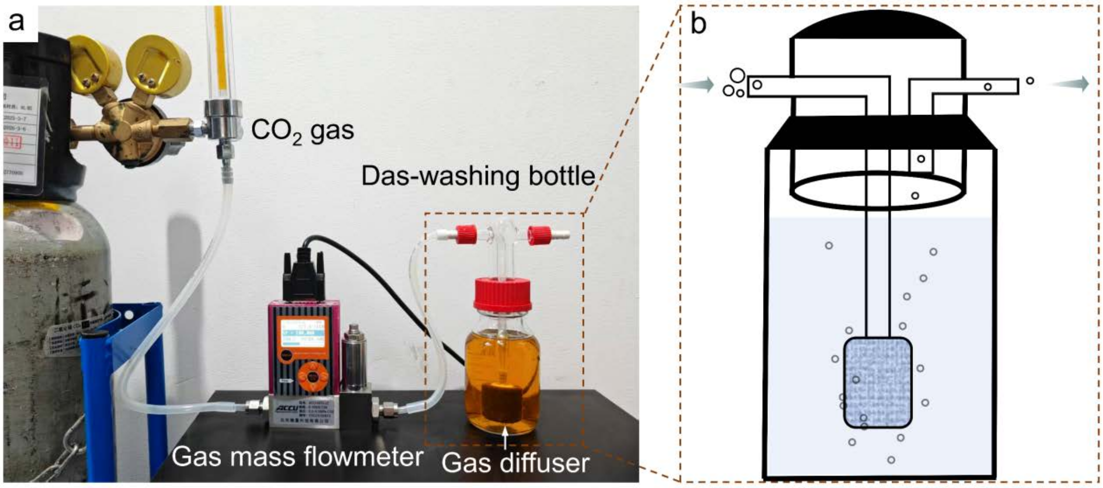

Figure S27. (a) The apparatus for CO_2 acidification of anolyte, (b) where CO_2 was introduced through a gas sparger to ensure efficient contact with the anolyte, with its flow rate regulated by a mass flow controller prior to introduction.

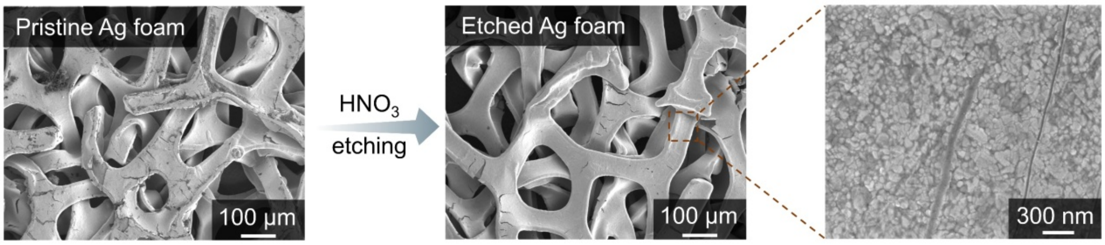

Figure S28. Surface SEM images of porous Ag electrode. The commercially purchased Ag foam was cleaned with nitric acid and deionized water to remove surface oxides and impurities.

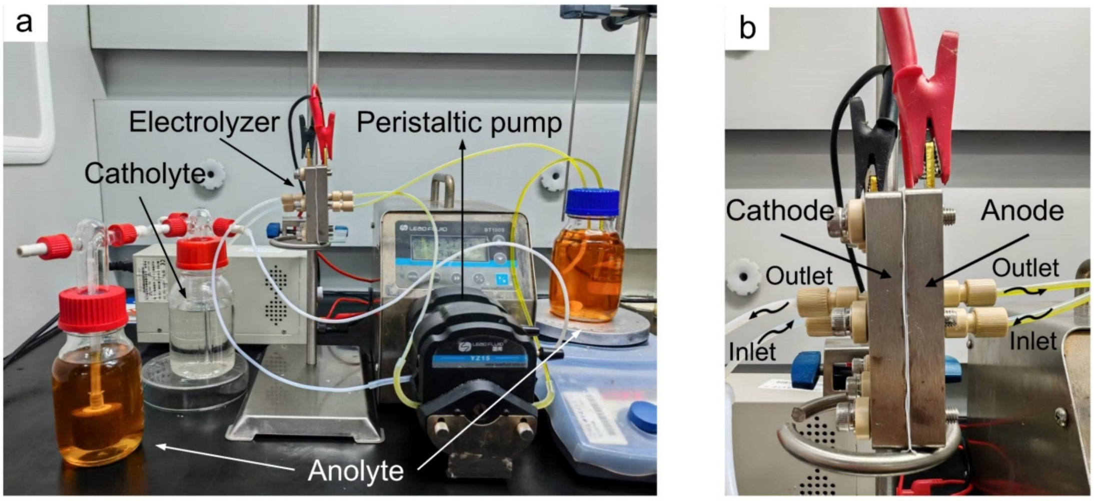

Figure S29. (a) Photograph of the self-sustaining paired electrolysis system. The cathode effluent gas is split via a dual-inlet buffer vessel, then directed into a scrubbing bottle equipped with a gas disperser for efficient gas–liquid extraction with the anolyte. (b) Photograph of the two-electrode electrolyzer. The electrolyte is fed through the bottom inlet and discharged from the top outlet at both anode and cathode.

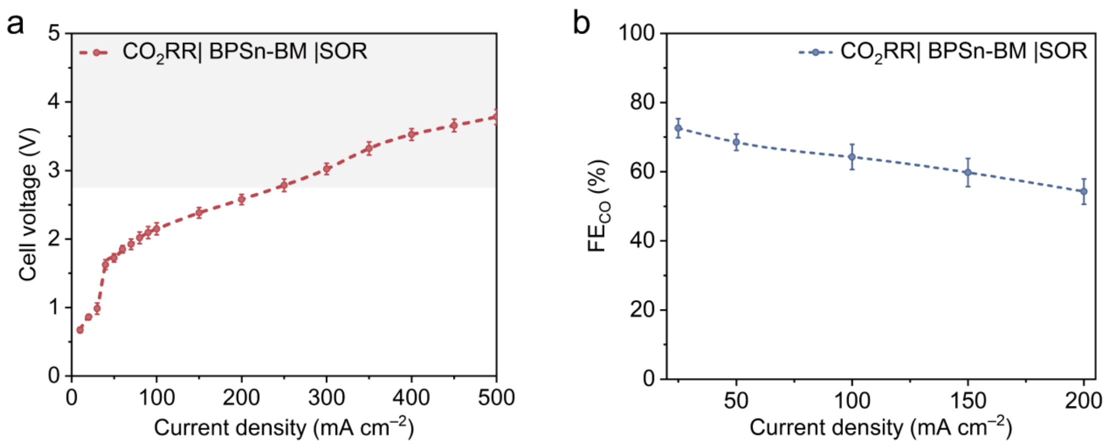

Figure S30. (a) Polarization curve of the self-sustained CO2RR| BPSn-BM |SOR electrolyzer using porous Ag foam cathode. (b) Current density-dependent FE_{CO} of the electrolyzer. The use of porous silver foam as the cathode did not significantly alter the electrolyzer polarization curve and CO Faradaic efficiency.

Supplementary Note 3: Water dissociation performance of the BPMs.

Given that carbon emission analysis is critically dependent on the grid scenario. A sensitivity analysis was conducted on the carbon emissions with respect to various energy sources, encompassing wind, solar, and thermal power, which bear carbon intensity of 0.01, 0.05, and 0.8 t_{CO2} MWh^{-1}, respectively.

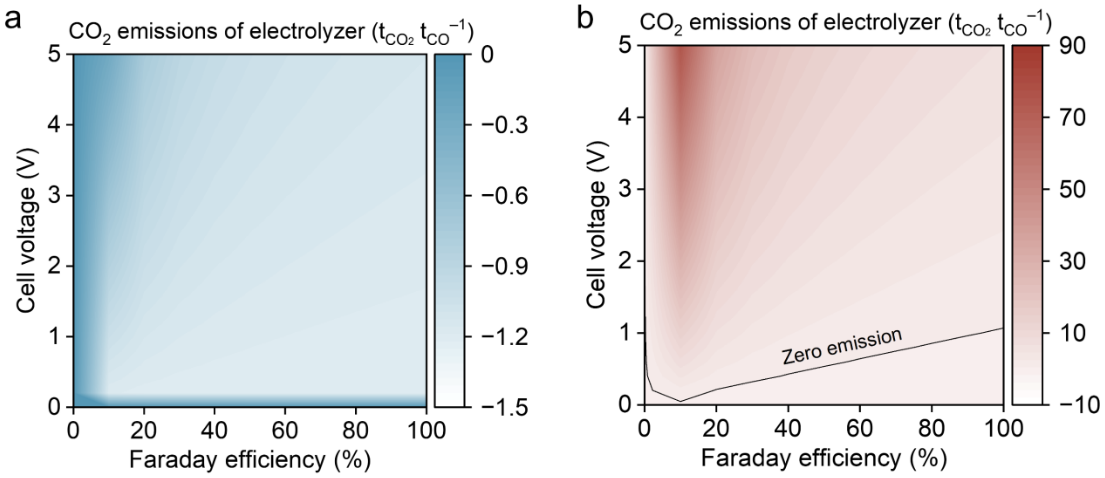

Figure S31. Carbon emission as a function of electrolyzer voltage and Faraday efficiency. (a) Employing wind power with a carbon intensity of 0.01 t_{CO2} MWh^{-1}. (b) Employing thermal power with a carbon intensity of 0.8 t_{CO2} MWh^{-1}.

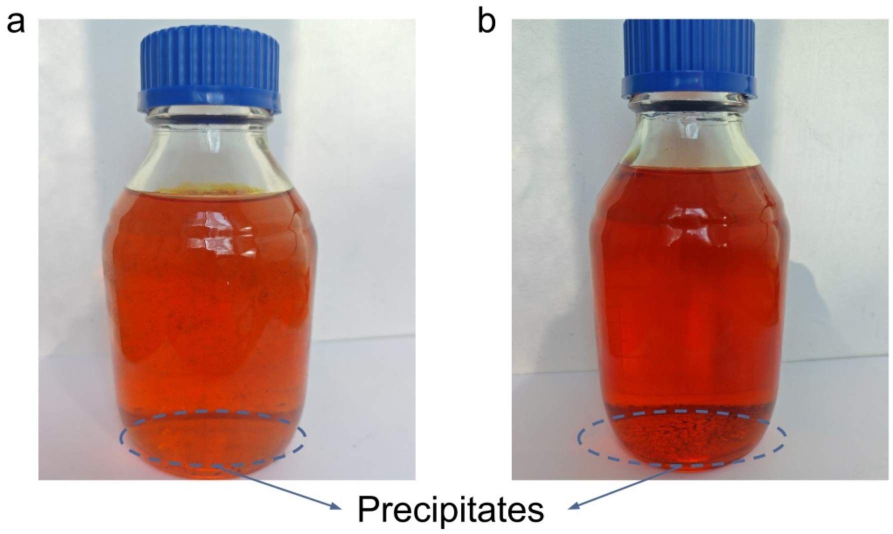

Figure S32. Photographs of the collected anolyte. Extended operation led to the formation of brown precipitates in the anolyte, indicating detachment of the anode catalyst from the porous electrode.

Supplementary Tables

Table S1. Parameters used in the COMSOL models.

Parameter	Value	Unit	Reference
Operating conditions			
T	298.15	T	–
P	1	atm	–
Model size			
$L_{electrode}$	200×10^{-6}	m	estimated
L_{PEM}	180×10^{-6}	m	estimated
L_{CEL}	20 (or 40) $\times 10^{-6}$	m	estimated
L_{AEL}	20 (or 40) $\times 10^{-6}$	m	estimated
Bulk concentrations			
$c_{HCO_3^-}$	3	M	estimated
c_{K^+}	3	M	estimated
c_{H^+}	$10^{-8.5}$	M	estimated
c_{OH^-}	$10^{-5.5}$	M	estimated
$c_{CO_2^{2-}}$	0.5×10^{-3}	M	estimated
c_{CO_2}	0.5×10^{-4}	M	estimated
Homogeneous reactions			
K_1	$10^{-6.37}$	M	[3]
k_1	0.03	s^{-1}	[3]
K_2	$10^{-10.32}$	M	[3]
k_2	59	s^{-1}	[3]
k_3	4.3×10^7	$m^3\ mol^{-1}\ s^{-1}$	[3]
k_4	4.8×10^5	$m^3\ mol^{-1}\ s^{-1}$	[3]
K_W	10^{-14}	M	[3]
Diffusion coefficient			
$D_{HCO_3^-}$	1.19×10^{-9}	$m^2\ s^{-1}$	[3]

D_{K^+}	1.90×10^{-9}	$m^2\ s^{-1}$	[3]
D_{H^+}	9.31×10^{-9}	$m^2\ s^{-1}$	[3]
D_{OH^-}	4.95×10^{-9}	$m^2\ s^{-1}$	[3]
$D_{CO_2{}^{2-}}$	8×10^{-10}	$m^2\ s^{-1}$	[3]
D_{CO_2}	1.66×10^{-9}	$m^2\ s^{-1}$	[3]

Table S2. Structure and water dissociation performance of BPMs.

BPMs	AEL/CEL matrices	WD catalysts	U_{100} (V)	Ref.
BPSn-BM	Quaternized-/Sulfonated-poly(terphenyl alkylene)	20 nm SnO_2	0.73	This work
FCBM	Quaternized-/Sulfonated-poly (phenylene oxide)	$Fe(OH)_3$ colloids	0.75	[8]
MBM	Quaternized poly (N-methyl-piperidine pterphenyl)/Perfluorinated sulfonic acid	30–50 nm SnO_2	0.76	[9]
3D-BPM	Quaternized-poly (phenylene oxide)/Sulfonated-PEEK	NH_2-MIL-101 MOF	0.88	[10]
Fe_2O_3@GO	Quaternized-polyethylene/ Sulfonated-PEEK	α-Fe_2O_3@GO	0.89	[11]
BPM-3D	Quaternized-polyethylene/	$Al(OH)_3$	0.95	[12]
BPM-2D	Sulfonated-PEEK	nanoparticles	1.2	
GO	PiperION A20/ Nafion NR 212	Commercial GO	0.97	[13]
3D BPM	Perfluorinated AEM/Nafion 211	GO	1.0	[14]
SCBM	Quaternized-poly (phenylene oxide)/Nafion 211	FeO(OH)	1.1	[15]
CIBM	Quaternized poly (N-methyl-piperidine -pterphenyl)/Poly (sulfonated-styrene-co-acrylonitrile)	SnO_2	1.1	[16]
4GO-BPM	Cross-linked AEL/Nafion	GO	1.45	[17]
Fe(III)@PEI BPM	Quaternized-poly (phenylene oxide)/Porous PAN	Fe(III)@PEI	1.8	[18]
PIL-BPM	Quaternized-poly (phenylene oxide)/Tianwei CEM	Polyaniline	1.87	[19]
FumaTech-FBM	–	–	1.04	Commercial
BPU	–	–	0.88	Commercial

Table S3. Reported Performance for CO_2 capture solution electrolyzers.

Current density (mA cm^{-2})	Cell voltage (V)	FE (%)	CO_2 utilization (%)	Stability (h)	Reference
100	2.16	55–65	> 97	300	This work
100	3.65	59	40	80	[20]
100	4.0	64	–	2	[21]
100	–	37	–	5	[22]
100	–	82	70	8	[23]
100	3.5	60	–	20	[24]
100	3.1	96	–	200	[25]
300	4.1	47 (C_{2+})	~ 100	25	[26]
100	3.7	75	35	24	[27]
200	3.7	93	65%	18	[28]
100	3.1	80	89	30	[29]
200	2.9	47	~ 100	40	[30]
100	2.7	70	~ 100	8	[31]
200	3.3	46	~ 100	20	[32]

References

1. Yu W, Zhang Z, Luo F *et al.* Tailoring high-performance bipolar membrane for durable pure water electrolysis. *Nat Commun* 2024; **15**: 10220.
2. Weng LC, Bell AT, Weber AZ. Towards membrane-electrode assembly systems for CO_2 reduction: a modeling study. *Energy Environ Sci* 2019; **12**: 1950–1968.
3. Lees EW, Bui JC, Song D *et al.* Continuum Model to Define the Chemistry and Mass Transfer in a Bicarbonate Electrolyzer. *ACS Energy Lett* 2022; **7**: 834–842.
4. Gallagher J. Compatible layers bring benefits. *Nat Energy* 2025; **10**: 10–10.
5. Simons R. Strong electric field effects on proton transfer between membrane-bound amines and water. *Nature* 1979; **280**: 824–826.
6. Chen L, Xu Q, Boettcher SW. Kinetics and mechanism of heterogeneous voltage-driven water-dissociation catalysis. *Joule* 2023; **7**: 1867–1886.
7. Blommaert MA, Vermaas DA, Izelaar B *et al.* Electrochemical impedance spectroscopy as a performance indicator of water dissociation in bipolar membranes. *J Mater Chem A* 2019; **7**: 19060–19069.
8. Ge Z, Shehzad MA, Yang X *et al.* High-performance bipolar membrane for electrochemical water electrolysis. *J Membr Sci* 2022; **656**: 120660.
9. Xu Z, Wan L, Liao Y *et al.* Continuous ammonia electrosynthesis using physically interlocked bipolar membrane at 1000 mA cm^{-2}. *Nat Commun* 2023; **14**: 1619.
10. Kwak D, Tran HM, Kandel DR *et al.* Synergistic effects of MOF catalysts and a 3D junction enable thin bipolar membranes to reach the thermodynamic minimum potential for water dissociation. *Chem Eng J* 2025; **507**: 160573.
11. Kim BS, Park SC, Kim DH *et al.* Bipolar Membranes to Promote Formation of Tight Ice-Like Water for Efficient and Sustainable Water Splitting. *Small* 2020; **16**: 2002641.
12. Shen C, Wycisk R, Pintauro PN. High performance electrospun bipolar membrane with a 3D junction. *Energy Environ Sci* 2017; **10**: 1435–1442.
13. Meng F, Qin J, Wu Q *et al.* Identifying the Critical Oxygenated Functional Groups on Graphene Oxide for Efficient Water Dissociation in Bipolar Membranes. *ACS Energy Lett* 2024; **9**: 5444–5451.

14. Chen Y, Wrubel JA, Klein WE *et al.* High-Performance Bipolar Membrane Development for Improved Water Dissociation. *ACS Appl Polym Mater* 2020; **2**: 4559–4569.
15. Shehzad MA, Yasmin A, Ge X *et al.* Shielded goethite catalyst that enables fast water dissociation in bipolar membranes. *Nat Commun* 2021; **12**: 9.
16. Xu Z, Liao Y, Pang M *et al.* A chemically interlocked bipolar membrane achieving stable water dissociation for high output ammonia electrosynthesis. *Energy Environ Sci* 2023; **16**: 3815–3824.
17. Yan Z, Zhu L, Li YC *et al.* The balance of electric field and interfacial catalysis in promoting water dissociation in bipolar membranes. *Energy Environ Sci* 2018; **11**: 2235–2245.
18. Ge Z, Shehzad MA, Ge L *et al.* Beneficial Use of a Coordination Complex As the Junction Catalyst in a Bipolar Membrane. *ACS Appl Energy Mater* 2020; **3**: 5765–5773.
19. Li G, Shehzad MA, Ge Z *et al.* In-situ grown polyaniline catalytic interfacial layer improves water dissociation in bipolar membranes. *Sep Purif Technol* 2021; **275**: 119167.
20. Zhang Z, Lees EW, Habibzadeh F *et al.* Porous metal electrodes enable efficient electrolysis of carbon capture solutions. *Energy Environ Sci* 2022; **15**: 705–713.
21. Li T, Lees EW, Zhang Z *et al.* Conversion of Bicarbonate to Formate in an Electrochemical Flow Reactor. *ACS Energy Lett* 2020; **5**: 2624–2630.
22. Li T, Lees EW, Goldman M *et al.* Electrolytic Conversion of Bicarbonate into CO in a Flow Cell. *Joule* 2019; **3**: 1487–1497.
23. Lees EW, Goldman M, Fink AG *et al.* Electrodes Designed for Converting Bicarbonate into CO. *ACS Energy Lett* 2020; **5**: 2165–2173.
24. Xing K, Wang M, Pan B *et al.* Efficient Bicarbonate Electrolysis to Formate Enabled via Ionomer Surface Modification in Cation Exchange Membrane Electrolyzers. *Angew Chem Int Ed* 2025; **64**: e202504835.
25. Zhang Z, Xi D, Ren Z *et al.* A carbon-efficient bicarbonate electrolyzer. *Cell Rep Phys Sci* 2023; **4**: 101662.
26. Lee G, Rasouli AS, Lee BH *et al.* CO_2 electroreduction to multicarbon products from carbonate capture liquid. *Joule* 2023; **7**: 1277–1288.
27. Kim Y, Lees EW, Berlinguette CP. Permeability Matters When Reducing CO_2 in an

Electrochemical Flow Cell. *ACS Energy Lett* 2022; **7**: 2382–2387.

28. Song H, Fernández CA, Choi H *et al.* Integrated carbon capture and CO production from bicarbonates through bipolar membrane electrolysis. *Energy Environ Sci* 2024; **17**: 3570–3579.
29. Nomoto K, Okazaki T, Beppu K *et al.* Highly selective formate formation via bicarbonate conversions. *EES Catal* 2024; **2**: 1277–1284.
30. Liu H, Shin H, Li XY *et al.* Hierarchically porous carbon supports enable efficient syngas production in electrified reactive capture. *Energy Environ Sci* 2025; **18**: 6628–6640.
31. Zhou B, Liu H, Su G *et al.* Electrosynthesis of CO from an electrically pH-shifted DAC post-capture liquid using a catalyst:support amide linkage. *Joule* 2025; **9**: 101883.
32. Xiao YC, Gabardo CM, Liu S *et al.* Direct carbonate electrolysis into pure syngas. *EES Catal* 2023; **1**: 54–61.
